# Photocatalytic Activity of Ionic Carbon Nitrides is Governed by Cation‐Modulated Dark Exciton Dynamics

**DOI:** 10.1002/advs.202509312

**Published:** 2025-08-21

**Authors:** Arindam Konar, Johannes Liessem, Changbin Im, Mohamed M. Elnagar, Dariusz Mitoraj, Pratibha Saini, Igor Krivtsov, Sarah Jasmin Finkelmeyer, Jan Griebel, Martin Presselt, Timo Jacob, Radim Beranek, Benjamin Dietzek‐Ivanšić

**Affiliations:** ^1^ Institute for Physical Chemistry Friedrich Schiller University Jena Lessingstrasse 4 07743 Jena Germany; ^2^ Leibniz Institute of Photonic Technology (Leibniz‐IPHT) Research Department Functional Interfaces Albert‐Einstein‐Strasse 9 07745 Jena Germany; ^3^ Institute of Electrochemistry Ulm University Albert‐Einstein‐Allee 47 89081 Ulm Germany; ^4^ Department of Chemical and Environmental Engineering University of Oviedo Oviedo 33006 Spain; ^5^ Center for Energy and Environmental Chemistry Jena (CEEC Jena) Friedrich Schiller University Jena Philosophenweg 7a 07743 Jena Germany; ^6^ Sciclus GmbH & Co. KG Moritz‐von‐Rohr‐Str. 1a 07745 Jena Germany; ^7^ Helmholtz‐Institute‐Ulm (HIU) Electrochemical Energy Storage 89081 Ulm Germany; ^8^ Karlsruhe Institute of Technology (KIT) 76021 Karlsruhe Germany; ^9^ Leibniz Institute of Surface Engineering (IOM) Permoserstraße 15 04318 Leipzig Germany

**Keywords:** carbon nitrides, dark excitons, photocatalysis, poly(heptazine imide), transient absorption spectroscopy

## Abstract

Ionic polymeric carbon nitrides, poly(heptazine imides) (PHIs), have emerged as promising photocatalysts, yet the relationship between their structure, exciton dynamics, and activity has remained elusive so far. Here, a direct link is established between photocatalytic activity, spectroscopic properties, and theoretical analysis of structural effects on exciton behavior in water‐soluble PHIs with different cations. Using steady‐state and time‐resolved emission spectroscopy alongside ultrafast transient absorption spectroscopy performed in the absence and presence of hole and electron quenchers, two distinct excitonic relaxation pathways are uncovered: sub‐100 ps decay dominated by exciton recombination and shallow‐trap states, and sub‐ns dynamics associated with deep‐trap assisted recombination. Notably, ethanol accelerated the sub‐100 ps decay via hole quenching, while the deep traps are unaffected by ethanol. The spectroscopic results show that the photocatalytic activity in H_2_O_2_ production (CsPHI << NaPHI < KPHI) correlates with exciton lifetimes, whereby the theoretical analysis reveals that the observed modulation of exciton lifetimes is primarily related to different dark exciton dynamics governed by changes in interlayer interactions due to the altered structural corrugation in the presence of various cations. This work establishes a unified structure–dynamics–activity relationship in PHIs, offering new design guidelines for PHI‐based photocatalytic materials.

## Graphical abstract

This study establishes a direct link between photocatalytic activity, spectroscopic properties, and theoretical analysis of structural effects on exciton behavior in ionic carbon nitrides with different cations. The exciton lifetimes and photocatalytic activity are shown to be primarily related to different dark exciton dynamics governed by changes in interlayer interactions due to the altered structural corrugation in the presence of various cations.

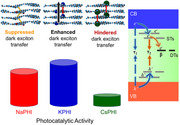

## 1 Introduction

To advance photocatalysis capable of mimicking natural photosynthesis and driving valuable chemical transformations, the development of low‐cost, scalable, and chemically stable photocatalytic materials is of paramount importance.^[^ ^1^ ^]^ Polymeric carbon nitrides (PCNs) represent a vigorously studied class of polymeric photocatalysts that attract attention particularly due to their low toxicity, high stability, and ease of processability.^[^ ^2^ ^]^ They also offer several advantages over classical semiconductors (e.g., TiO_2_), such as a red‐shifted optical absorption edge toward the visible light region (bandgap of ≈2.7–2.9 eV) and exceptional activity in important chemical conversions (e.g., photocatalytic H_2_O_2_ production from O_2_).^[^ ^3^ ^]^ PCNs can be obtained using facile synthesis by thermal polymerization from inexpensive nitrogen‐rich precursors such as melamine, urea, thiourea, cyanamide, dicyandiamide, whereby the resulting structural, photophysical, and surface‐catalytic properties often depend heavily on the synthetic route employed.^[^ ^2^, ^4^ ^]^ In contrast to conventional (non‐ionic) PCNs, poly(heptazine imide) (PHI)‐based carbon nitrides represent a distinct subclass of *ionic* carbon nitrides comprising stacks of negatively charged 2D heptazine‐based frameworks counterbalanced by alkali metal cations and/or protons.^[^ ^3^, ^5^ ^]^ Notably, the presence of surface functional groups in PHIs, such as cyanamide and cyamelurate moieties, leads to improved wettability and dispersibility, which often renders ionic carbon nitrides more photocatalytically active compared to conventional PCNs.^[^ ^3^, ^5^ ^]^ Moreover, in contrast to conventional PCNs, PHI‐based ionic carbon nitrides have also shown the capability to accumulate and store photogenerated electrons in long‐lived trap states, which has been demonstrated to enable delayed (“dark”) photocatalysis and photodoping phenomena.^[^ ^6^ ^]^ It is well‐established that the nature of cations in ionic carbon nitrides exerts a significant effect on their photophysical and surface catalytic properties, and typical strategies to tune the photocatalytic performance of PHIs include efforts to control the materials properties by choice of different cations or cation combinations during synthesis.^[^ ^3^, ^7^ ^]^

In general, spectroscopic investigations of (both conventional and ionic) PCNs have proven to be a powerful tool to study the photophysics of the materials. Merschjann et al. investigated conventional PCN using transient absorption spectroscopy (TAS) and proposed that initially excited singlet excitons rapidly dissociate into singlet polaron pairs and subsequently into free polarons. The latter then undergoes interlayer out‐of‐plane diffusion followed by recombination and (non)radiative relaxation, thereby characterizing exciton‐mediated charge transfer in 1D carbon nitride melon as an interlayer conduction mechanism.^[^ ^8^ ^]^ In a similar manner, Noda et al. combined time‐resolved spectroscopic techniques with direction‐dependent electrical measurements to determine the charge carrier mobilities in triazine‐based PCN films, which revealed a higher out‐of‐plane conductivity compared to in‐plane.^[^ ^9^ ^]^ Using ultrafast time‐resolved spectroscopy, Durrant et al. observed that cyanamide surface‐functionalized PHI‐type PCN is capable of accumulating ultralong‐lived trapped electrons for fuel generation in the dark, whereby the charge recombination becomes ≈400‐times faster due to the accumulation of the long‐lived electron after hole extraction.^[^ ^10^ ^]^ Notably, systematic investigations of the role of various cations in ionic PCNs are rather scarce. Kröger et al. addressed the relation between the ionic conductivity and photocatalytic activity in the structural pores of metal‐PHIs by studying the influence of various counterions, e.g., Li^+^, Na^+^, K^+^, Cs^+^, Ba^2+^, NH_4_^+^, and tetramethyl ammonium.^[^ ^7a^ ^]^ Recently, Pan et al. reported various PHI‐based photocatalysts modified with co‐catalysts for overall water splitting and proposed that, depending on cation size, the different interlayer stacking modes in PHI can promote exciton diffusion and prolong the photoexcited electron lifetimes.^[^ ^7b^ ^]^ However, our knowledge of the cation‐dependent relationship between the structure, exciton dynamics, and activity in PHIs has remained limited so far, which makes the rational design of high‐performance PHI‐based photocatalysts very challenging.

Herein, we employ steady‐state and time‐resolved emission spectroscopy alongside ultrafast transient absorption spectroscopy to study excitonic dynamics in three different model water‐soluble PHI photocatalysts (Na‐, K‐, and CsPHI) which exhibit different activities in light‐driven H_2_O_2_ production. Importantly, the photocatalytic H_2_O_2_ production from oxygen‐containing aqueous alcoholic solutions is well‐established as one of the most important conversions photocatalyzed by PHIs,^[^ ^3^, ^11^ ^]^ and offers the advantage to study the fundamental excitonic dynamics in PHIs without any undesired interference from additional co‐catalysts. In addition, the use of water‐soluble PHIs,^[^ ^3b^ ^]^ i.e., optically transparent aqueous solutions containing fully dispersible PHI nanoparticles, enables overcoming detrimental issues typically encountered in spectroscopic studies of optically scattering suspensions.^[^ ^6^, ^12^ ^]^ Most notably, we provide a direct link between our spectroscopic results and our recent theoretical studies, in which the photocatalytic activity of PHI‐based ionic carbon nitrides is proposed to be crucially influenced by dark exciton (i.e., momentum‐forbidden exciton) dynamics.^[^ ^13^ ^]^ This enables us to establish, for the first time, a direct and consistent correlation between photocatalytic activity, spectroscopic properties, and theoretical analysis of structural effects on exciton behavior in PHIs with different cations, which will provide essential guidelines for further development of PHI‐based photocatalysts.

## 2 Results and Discussion

**Figure 1:**
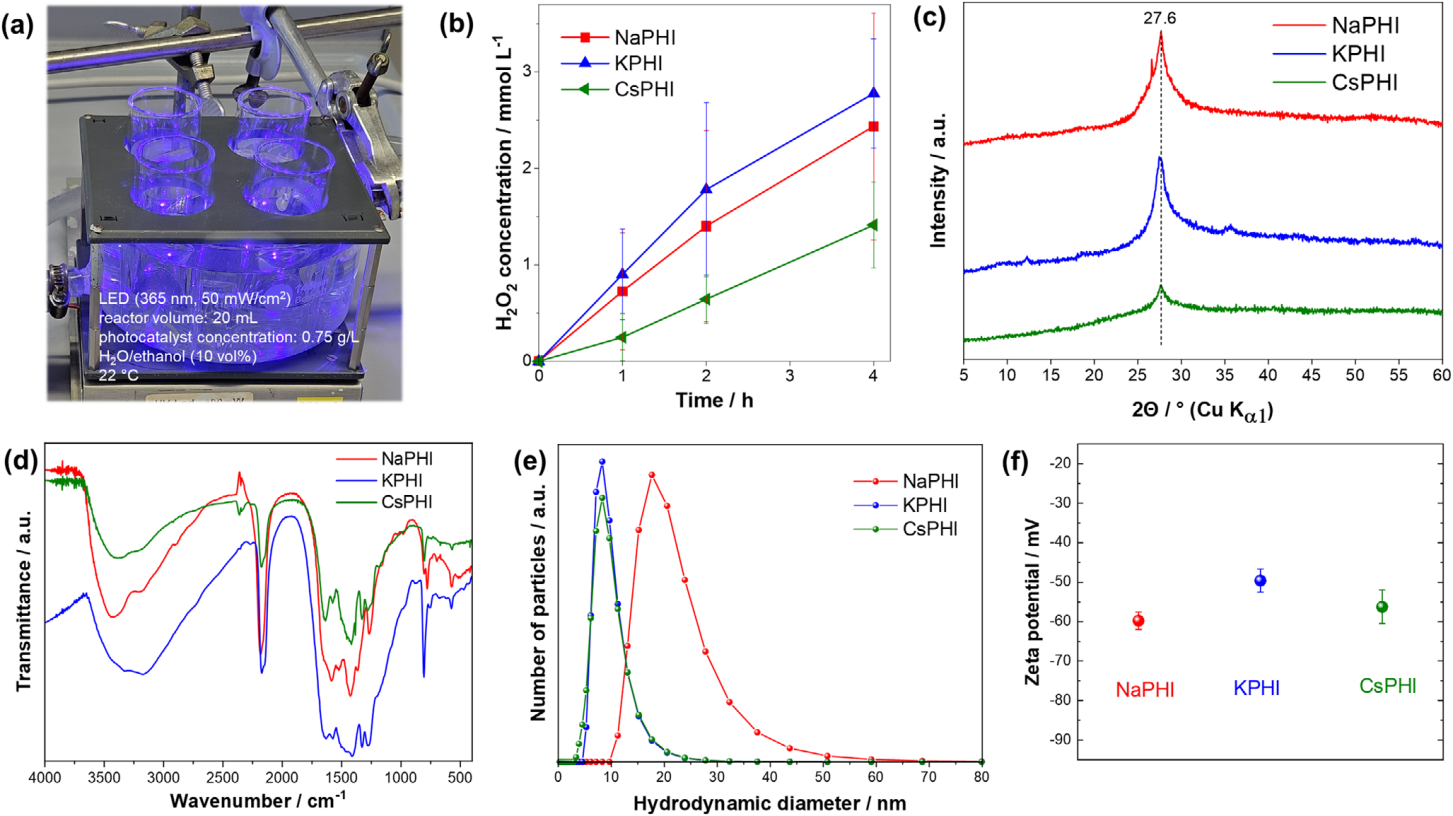
a) Photocatalytic H_2_O_2_ evolution in UV LED (365 nm) irradiated reactors containing PHI photocatalyst in ethanolic aqueous solution (10 vol.% EtOH) stirred under air. b) Photocatalytic performance of water‐soluble PHI photocatalysts containing different cations. Further characterization of NaPHI, KPHI, and CsPHI samples: c) X‐ray diffraction patterns, d) FTIR spectra, e) particle size distributions obtained from the dynamic light scattering measurements, and f) zeta potential values of native PHI solutions after dialysis at pH∼8. Standard errors were calculated from at least three experiments, and error bars represent the 95% confidence intervals.

Synthetic parameters of the photocatalysts are given in Table S1 (Supporting Information). Energy Dispersive X‐ray Spectroscopy (EDS) and elemental mapping analyses confirm the homogeneous distribution of alkali metal cations within the PHI materials, as shown in Figures S1–S3 (Supporting Information). The atomic percentages of the respective cations are comparable across samples: 4.7±0.1% Na in NaPHI, 4.4±0.2% K in KPHI, and 5.5±0.8% Cs in CsPHI. These results indicate a uniform cation distribution and consistent loading levels, confirming the effective incorporation of alkali metals into the PHI framework. All studied water‐soluble PHI photocatalysts exhibit significant photocatalytic activity in H_2_O_2_ evolution from air‐containing ethanolic aqueous solutions under UV (365 nm) LED irradiation (**Figure** **1**a,b). The photocatalytic activity increased in the order CsPHI << NaPHI < KPHI, whereby the initial H_2_O_2_ evolution rates (taken after 1 h) were 0.25 ± 0.06 mmol L^−1^ h^−1^, 0.73 ± 0.14 mmol L^−1^ h^−1^ and 0.90 ± 0.19 mmol L^−1^ h^−1^ for CsPHI, NaPHI and KPHI, respectively. Notably, the observed trend in photocatalytic activity of the samples cannot be correlated in any straightforward way to any trends observed in the characterization of their basic structural, morphological, and surface properties. The X‐ray diffraction (XRD) patterns of all samples recorded after dialysis and drying exhibit typical characteristics of PHI materials, with no significant shifts of peak positions between the materials containing different cations (Figure 1c). However, the broad peaks at ≈27.6° (3.2 Å) corresponding to the (001) crystal plane of PHI^[^ ^5d^ ^]^ are differently pronounced for different PHIs, suggesting different degrees of crystallinity and variations of interplanar distances in the three PHI materials containing different cations. Yet, these differences are difficult to quantify due to the low signal‐to‐noise ratio of the XRD data. The sharp reflex at 26.6° in the XRD pattern of NaPHI is due to traces of non‐condensed melamine from the synthesis. The Fourier‐transform infrared (FTIR) spectra recorded after dialysis and drying are also typical for PHI materials with no appreciable differences between NaPHI, KPHI, and CsPHI samples (Figure 1d). They exhibit a fingerprint region at 1140–1700 cm^−1^, ascribed to stretching vibrations corresponding to ν(C–N) and ν(C═N), characteristic of PCN materials. The prominent peak at ≈800 cm^−1^ is attributed to the triazine ring breathing mode, while the signal at 3420 cm^−1^ is associated with the stretching vibrations of secondary and primary amines and also hydroxide groups incorporated into the PHI structure as a result of the synthesis in the alkali melt. The signals observed between 2100 and 2200 cm^−1^, corresponding to ν(C≡N), appear typically in the FTIR spectra of samples synthesized in molten salts, but are absent in conventional (non‐ionic) carbon nitrides. The mean hydrodynamic diameters of the photocatalytically most active (KPHI) and least active (CsPHI) samples were nearly identical (8.3 ± 5.4 nm and 8.3 ± 5.9 nm), whereas the mean hydrodynamic diameter of the NaPHI sample was 20.5 ± 13.0 nm (Figure 1e). The differences of the zeta potentials of all samples, i.e., the electric (Galvani) potentials at the shear plane of the particles with respect to the electric potential in the bulk solution, were also negligible, −60 ± 1 mV, −50 ± 1 mV and −56 ± 2 mV at pH ≈8 for NaPHI, KPHI and CsPHI samples, respectively (Figure 1f).

The fact that conventional investigations of structural, morphological and surface properties could not account directly for the stark differences in their photocatalytic performance prompted us to investigate the photophysics and charge carrier dynamics of NaPHI, KPHI and CsPHI samples in detail using various spectroscopic techniques at different time scales and upon the addition of various additional reducing and oxidizing agents acting as chemical quenchers.

### 2.1 Steady‐State and Time‐Lapse UV–vis Absorption

**Figure 2:**
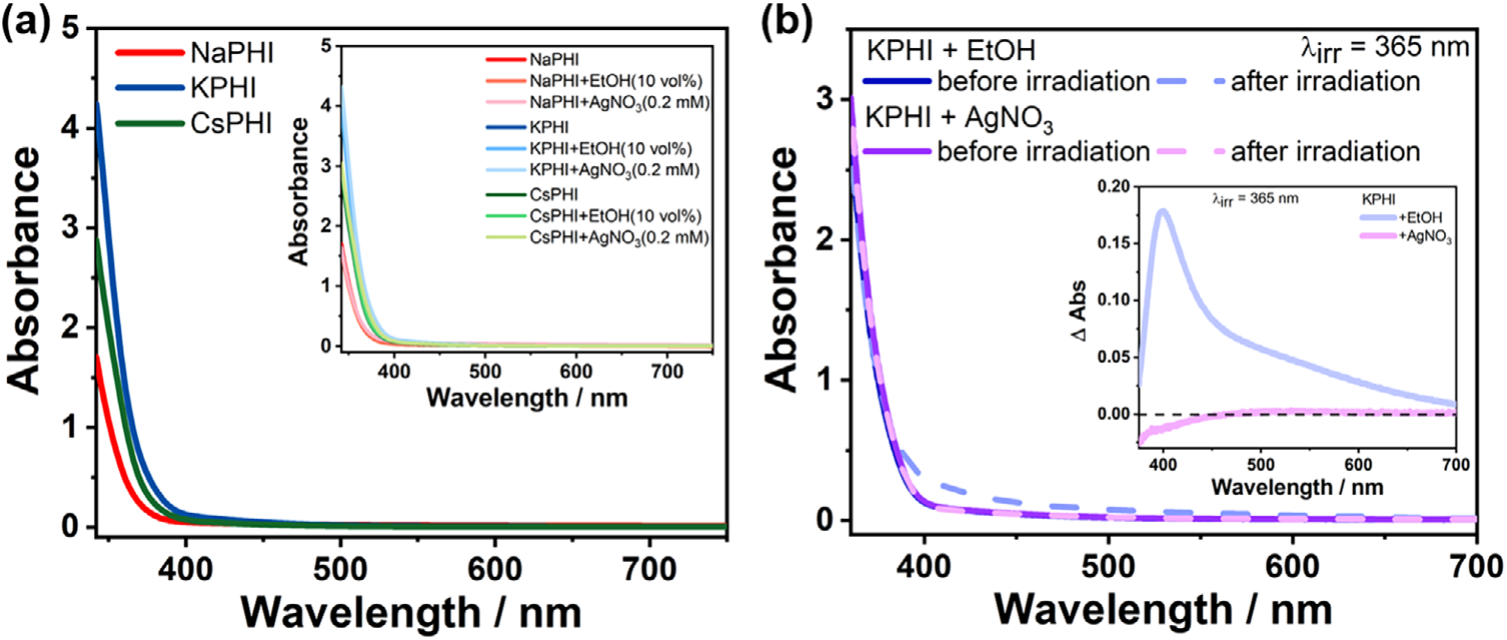
a) Steady state UV–vis absorption spectra of Na‐, K‐, and CsPHI (concentration of 1 g L^−1^). Inset corresponds to the absorption spectra of all the PHIs in the presence of EtOH (10 vol.%) and AgNO_3_ (0.2 mM). b) UV–vis absorption spectra of KPHI in the presence of 10 vol.% EtOH and 0.2 mM AgNO_3_ before (solid lines) and after (dashed lines) 2 h of 365 nm LED irradiation. The inset shows the corresponding absorption difference spectra.

As the first step, steady‐state UV–vis absorption spectra were recorded from aqueous suspensions of the water‐soluble PHI samples at a concentration of 1 g L^−1^, either without or with addition of ethanol (EtOH) as a sacrificial hole scavenger or silver nitrate (AgNO_3_) acting as an electron quencher after electronic excitation of the PHI. **Figure** **2**a shows the steady‐state absorption spectra of Na‐, K‐, and CsPHI. All PHIs show an absorption edge at 400 nm, without any appreciable absorption in the visible range. Addition of either 10 vol.% EtOH or 0.2 mM AgNO_3_ does not alter the absorption behavior of any of the PHIs, as shown in the inset of Figure 2a. Two further methods have been employed to study the electronic absorption properties of our materials. First, photothermal deflection spectroscopy (PDS) was used to measure thin (≈400–500 nm) films on glass obtained from water‐soluble PHI samples using gelation and calcination according to established methodologies.^[^ ^14^ ^]^ Since PDS measures the absorption by detecting the thermalized energy of the absorbed photons and not the transmitted light, it is not affected by scattering and is highly sensitive to weak absorption.^[^ ^15^ ^]^ For these thin (≈400–500 nm) films, the PDS data of all three samples exhibit similar absorption behavior as in solution, exhibiting an absorption edge in the near‐visible range at ≈430 nm (see Figure S4a, Supporting Information). Second, thicker films were obtained by conventional drop‐casting the water‐soluble PHIs onto glass substrates, and the electronic absorption properties were characterized using both UV–vis diffuse reflectance spectroscopy (DRS) and PDS. The UV–vis DRS spectra revealed an absorption shoulder at ≈430 nm as well as a long and weaker absorption tail down to 550 nm (see Figure S5a, Supporting Information), with these sub‐bandgap features being most pronounced in the case of Cs‐PHI. Comparable spectral features were obtained for these drop‐cast films using PDS (see Figure S4b, Supporting Information). The emergence of these sub‐bandgap visible‐light absorption features, which were not detectable in the spectra recorded in aqueous suspensions or very thin films, can be rationalized by the significantly increased optical path length when measured on drop‐cast solid films on glass substrates, thus enhancing the detection sensitivity for weak absorption bands. Given that such sub‐bandgap absorption bands and tails typically correlate with structural disorder and defect states in carbon nitrides,^[^ ^16^ ^]^ these results might suggest that the inferior photocatalytic performance of CsPHI might originate from its higher structural disorder,^[^ ^17^ ^]^ as suggested also by the less pronounced XRD reflexes (see Figure 1c).

In addition, the changes in absorption of the PHIs upon 2‐h irradiation with an LED at 365 nm in the presence of either 10 vol.% EtOH or 0.2 mM AgNO_3_ were monitored by time‐lapse steady‐state UV–vis absorption spectroscopy (see Figure 2b; Figure S5b, Supporting Information). In the presence of 10 vol.% EtOH, a broad absorption tail between 380 and 650 nm emerges for KPHI after illumination (Figure 2b).

The changes induced by irradiation in the presence of the hole quencher are more clearly reflected in the differential absorption spectrum, which shows a broad band between 450 and 700 nm and a maximum ≈400 nm (see Figure 2b). This indicates efficient reductive quenching of the electronically excited KPHI^*^ state by EtOH and the formation of KPHI^●–^ anionic radicals, as evidenced by EPR spectroscopy (Figure S6, Supporting Information). No continuously increasing accumulation of the radical species is observed since the KPHI^●–^ radical anion formed clearly reacts with oxygen dissolved in the solution, a reaction which is known to occur readily.^[^ ^6e^ ^]^ In contrast, irradiation of the sample in the presence of 0.2 mM AgNO_3_ leaves the absorption of the sample unaltered (see Figure 2b), a behavior that is similarly observed for NaPHI and CsPHI (Figure S5b,c, Supporting Information). This indicates that AgNO_3_ can effectively scavenge the photogenerated electrons, either directly or via fast reaction with KPHI^●–^ radical anions. Irradiation of NaPHI and CsPHI in the presence of EtOH leads to similar, albeit less pronounced, absorption changes as for KPHI (Figure S5b,c, Supporting Information). This confirms the generation of radical anions at Na‐, and CsPHI in the presence of EtOH, yet with a diminished rate as compared to KPHI. This can be explained either by a relatively higher rate of exciton recombination and/or faster quenching of the radical anion by dioxygen than in the case of KPHI.

### 2.2 Steady‐State and Time‐Resolved Emission

**Figure 3:**
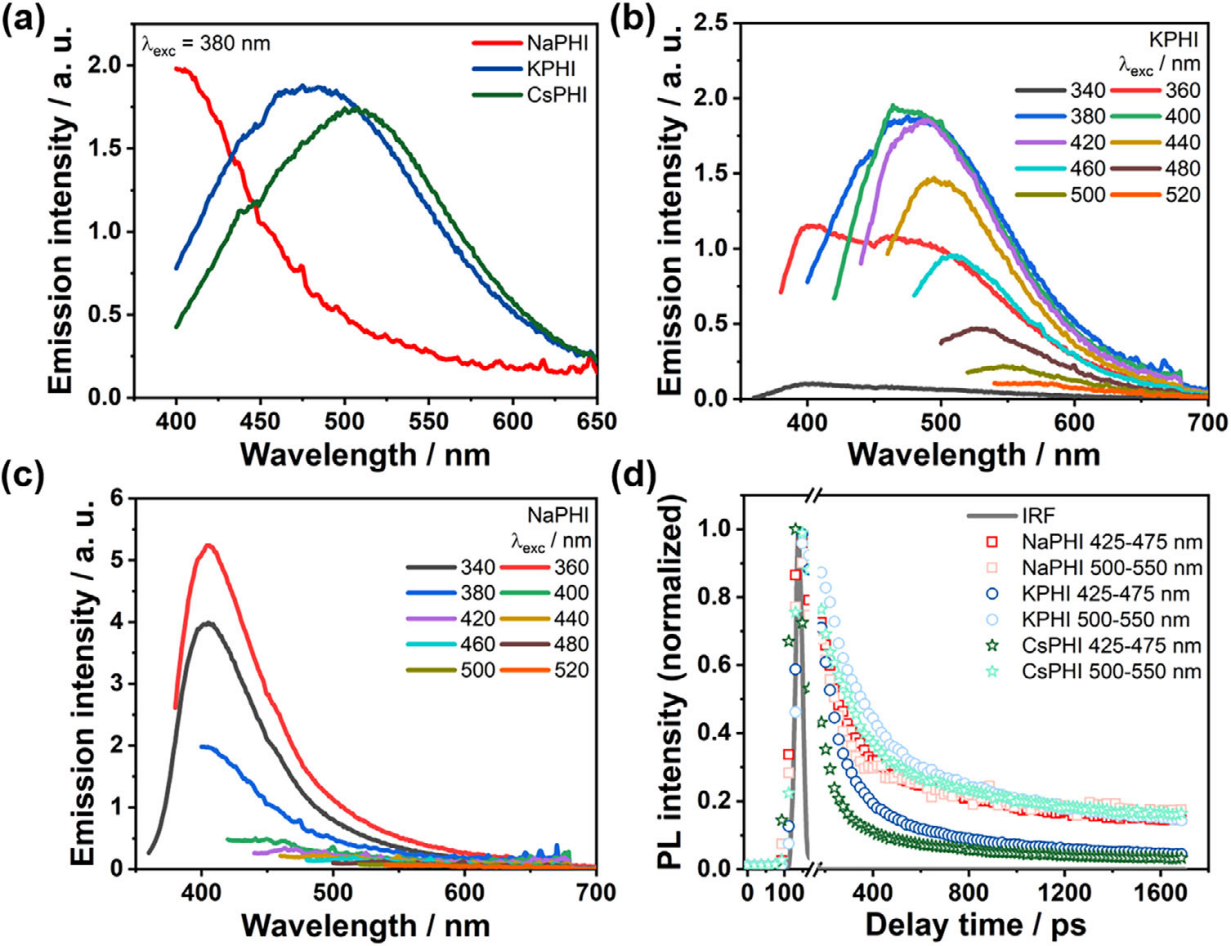
a) Steady‐state emission spectra of Na‐, K‐, and CsPHI (concentration 1 g L^−1^) excited at 380 nm. b) Steady‐state emission spectra of KPHI (concentration 1 g L^−1^) with various excitation wavelengths. Optical densities at excitation wavelengths vary from 0.1 (λ_exc_ = 340 nm) to 0.0003 (λ_exc_ = 520 nm). c) Steady‐state emission spectra of NaPHI (concentration 1 g L^−1^) with excitation wavelengths varying from 340 to 520 nm. Optical densities at excitation wavelengths vary from 0.1 (λ_exc_ = 340 nm) to 0.001 (λ_exc_ = 520 nm). d) Comparison of time‐resolved emission decay kinetics of NaPHI, KPHI, and CsPHI (1 g L^−1^ concentration) at different spectral ranges upon excitation at 370 nm. Instrumental response function (IRF) is given as the gray line.

Interestingly, the steady‐state emission spectra of the PHIs (**Figure** **3**a) indicate a redshift of the emission maxima with increasing cation size (Na < K < Cs). Upon excitation at 380 nm, NaPHI shows maximum emission at 405 nm, while the emission maxima get shifted to longer wavelengths to 475 and 508 nm for KPHI and CsPHI, respectively. Furthermore, KPHI exhibits an excitation wavelength‐dependent emission maximum (Figure 3b). Upon shifting the excitation wavelength from 340 to 520 nm, the emission maximum shifts from 405 to 585 nm. Such excitation wavelength‐dependent emission has not been observed in conventional carbon nitrides synthesized by thermal polycondensation of melamine; these materials typically show a narrow emission band, in contrast to KPHI.^[^ ^6^, ^18^ ^]^ Nonetheless, excitation wavelength‐dependent emission is not uncommon for metal oxide photocatalysts, e.g. ZnO, in which various trap states contribute to different emission characteristics.^[^ ^19^ ^]^ Thus, we assume that, during the ionothermal synthesis of the PHIs, various types of states related to structural features and surface functionalization arise, which act as local traps with distinct optical characteristics. As these different emissive species contribute to the overall emission, they result in the rather broad overall emission band of KPHI.

Similar excitation wavelength‐dependent emission is observed for CsPHI (see Figure S7a, Supporting Information). In contrast, the emission of NaPHI appears to be independent of the excitation wavelength (Figure 3c). We suppose that this relates to the small size of the Na cation, which prevents the exfoliation of the heptazine‐based PHI layers, and to the overall larger particle size of NaPHI as compared to KPHI and CsPHI (see Figure 1e), which both result in diminished impact of surface‐related traps on the emission properties. Consequently, the rather narrow and excitation wavelength‐independent emission of NaPHI is observed (Figure 3c).

Time‐resolved emission studies of KPHI follow the decay of the broad emission band (Figure S7b, Supporting Information) in the range of 420–550 nm upon excitation at 370 nm. More specifically, we compare nanosecond (ns) emission kinetics integrated in the range between 425 and 475 nm as well as between 500 and 550 nm (Figure 3d). The 425–475 nm decay kinetics reveal a characteristic half‐lifetime of 0.22 ns, while the emission decay observed between 500 and 550 nm appears to be slightly slower, corresponding to a half‐lifetime of 0.36 ns. This difference in lifetimes corroborates the presence of two distinct emissive states, likely associated with defect‐bound excitons, as also inferred from the excitation wavelength‐dependent steady‐state emission measurements (Figure 3b). The mobility of the excitons in deeper traps is typically limited, hence resulting in the slightly slower decay of the emission at longer wavelengths.

Following the same protocol, our analysis of the emission decay kinetics for CsPHI revealed that the emission decay probed between 425 and 475 nm, as well as between 500 and 550 nm, is characterized by a half‐lifetime of 0.17 ns and 0.30 ns, respectively. NaPHI, on the other hand, shows only a single characteristic decay constant of ≈0.24 ns, across the range of emission wavelengths (Figure 3d). This provides further indication that the choice of cation modulates the overall surface trap density in the PHIs, whereby the overall larger particle size of NaPHI (see Figure 1e) and the inability of the small Na cation to effectively exfoliate the PHI layers result in a lower concentration of surface defect‐related excitonic states.

### 2.3 Ultrafast Transient Absorption Spectroscopy

**Figure 4:**
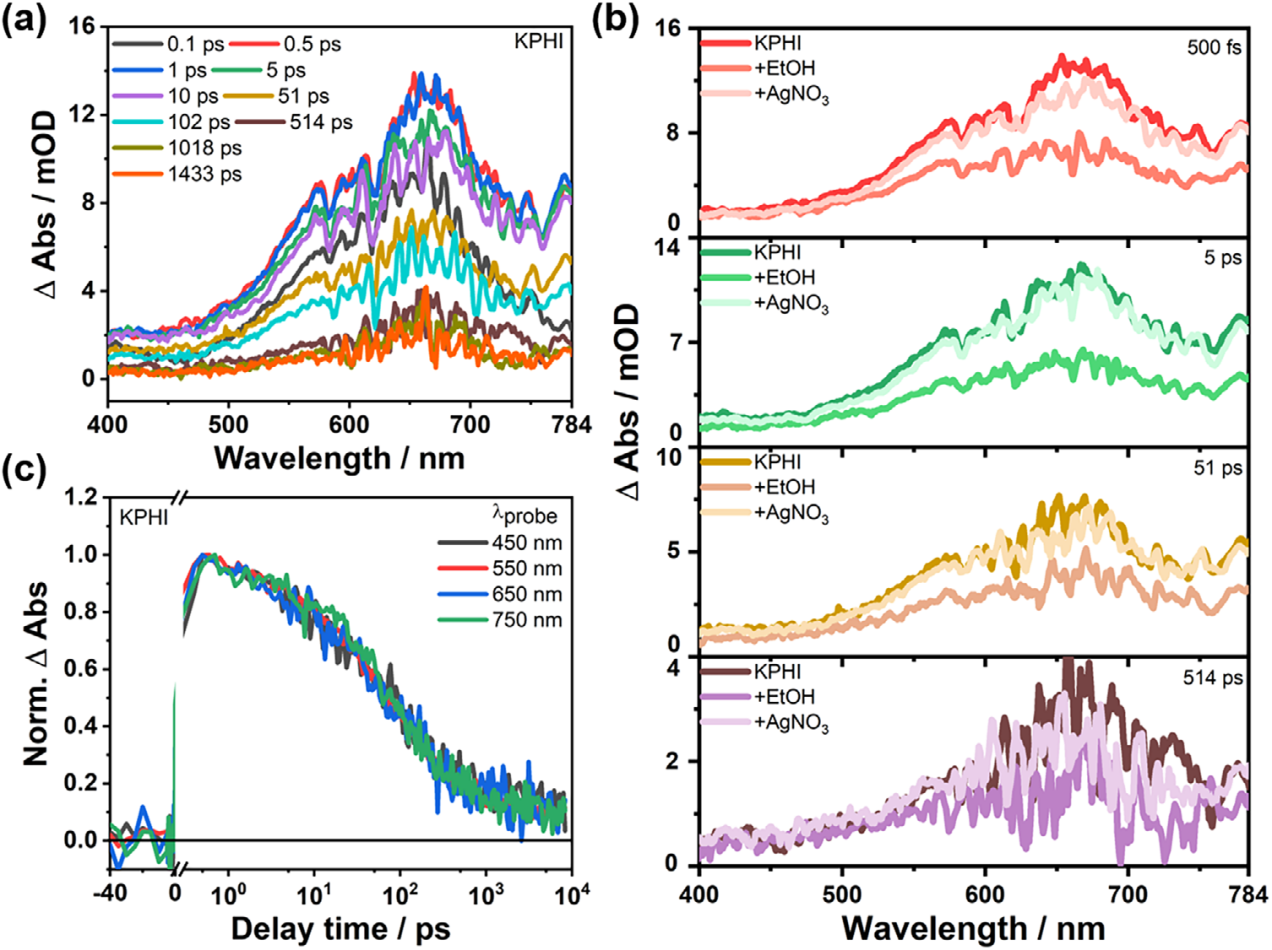
a) fs‐transient absorption spectra of KPHI (concentration 1 g L^−1^) measured at selected time delays after excitation at 325 nm, b) fs‐transient absorption spectra of KPHI (concentration 1 g L^−1^) at selected time delays in presence of EtOH (10 vol.%) and AgNO_3_ (0.2 mM) after excitation at 325 nm, c) Normalized decay kinetics of KPHI (concentration 1 g L^−1^) at selected wavelengths of 450, 550, 650, and 750 nm.

We first investigated the ultrafast photoinduced dynamics of KPHI by fs‐TA spectroscopy upon excitation at 325 nm. The corresponding spectra (**Figure** **4**a) feature a broad photoinduced absorption centred at 665 nm. Such broad and unstructured spectral features, not uncommon also in inorganic semiconductors, make it challenging to disentangle the evolution of the photogenerated species (e.g., excitons, trapped or free electrons or holes).^[^ ^20^ ^]^ Nonetheless, the different contributions can be sorted out by studying the PHI in the presence of chemical quenchers, e.g. EtOH or AgNO_3_. Although the photoinduced absorption behavior in the visible region is not drastically altered in the presence of both quenchers, the intensity of the TA signal gets reduced in the presence of 10 vol.% EtOH, while the quenching is much less prominent upon the addition of 0.2 mM AgNO_3_ (Figure 4b). Similar observations were made for Na‐ and CsPHI in the presence of both quenchers (Figure S8, Supporting Information).

Due to rather small spectral‐temporal changes (within the experimentally accessible signal‐to‐noise ratio) of the TA data, we spectrally integrated the TA features in the visible region between 450 and 750 nm. The resulting kinetics reflect the overall decay of the excited state absorption (ESA).^[^ ^6^, ^10^, ^20^, ^21^ ^]^ Furthermore, TA kinetics at single probe wavelengths, i.e., at 450, 550, 650, and 750 nm, are considered (Figure 4c). However, no significant changes in the kinetics are observed upon variation of the probe wavelengths. This finding implies that the different trap states do not contribute significantly differently on the ps‐ to ns‐time scale of the TA measurement.

### 2.4 Transient Absorption in the Absence of Quenchers

**Figure 5:**
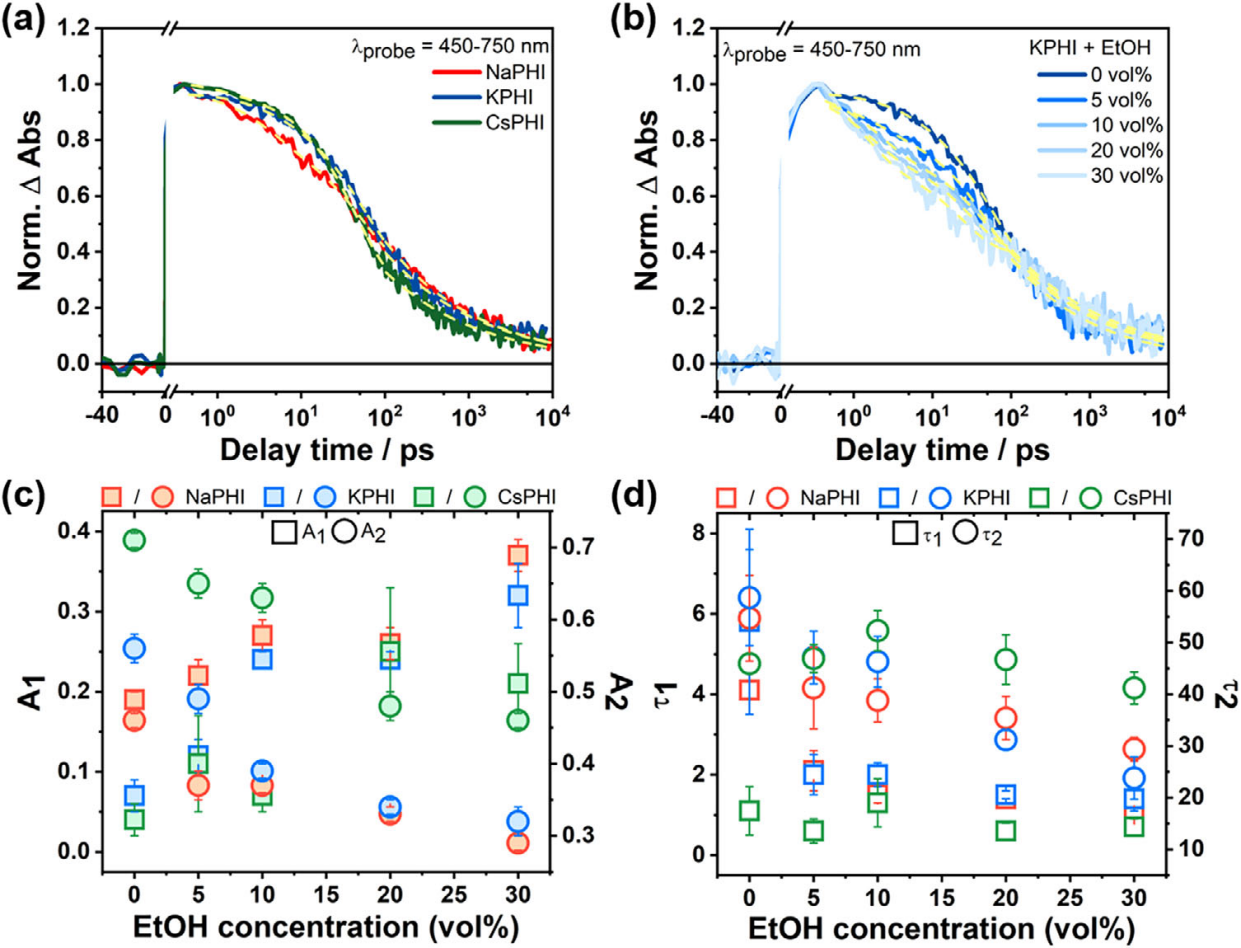
a) Decay kinetics of Na‐, K‐, and CsPHI (concentration 1 g L^−1^) obtained by integrating the TA signal in the probe wavelength range from 450 to 750 nm. The yellow dashed lines represent the fit to the data utilizing the fit function described in the text. b) Charge recombination kinetics of KPHI (concentration 1 g L^−1^) probed from 450 to 750 nm with variation of EtOH volume fraction from 0 to 30 vol.%. Yellow dashed lines represent biexponential and power law fitting. c) Change of amplitudes (*A*_1_ and *A*_2_) obtained from the biexponential fit within 100 ps of KPHI (concentration 1 g L^−1^) with varying EtOH concentrations. d) Change of characteristics time constants (τ_1_ and τ_2_) obtained from the biexponential fit within 100 ps of KPHI (concentration 1 g L^−1^) with varying EtOH concentrations. Each sample was measured in triplicate, and the error bars are given as ±σ (σ is the standard deviation of the fitted parameter obtained from the nonlinear regression using OriginLab software).

The TA data for NaPHI and CsPHI resemble those of KPHI upon excitation at 325 nm (see Figure S9a,b, Supporting Information), both in terms of the spectral shape and the position of the ESA bands as well as the excited‐state decay kinetics as reflected in the single‐wavelength kinetics (**Figure** **5**a; Figure S9c,d, Supporting Information).

Concentration‐dependent fs‐TA experiments were also carried out for the different PHIs with concentrations ranging from > 1.0 to 0.1 g L^−1^ (Figure S10, Supporting Information). However, the decay kinetics appear unaltered upon varying the concentration, irrespective of the cations in the material. This suggests that the aggregation‐induced energy transfer between PHI particles or other aggregation effects is not significant on the timescale probed in the TA experiments.

The quantitative analysis of the TA kinetics relies on a model previously proposed by Li et al.,^[^ ^6^, ^12^ ^]^ suggesting geminate recombination of a single exciton pair within a particle on an early time scale modelled by an exponential function, and trap‐assisted recombination at relatively long delay times modelled by a power law.^[^ ^6^, ^10^, ^21^ ^]^ The early TA kinetics of KPHI, i.e., up to a delay time of 100 ps, can be well‐fitted by $I=A_{1}e^{-t/\tau_{1}}+A_{2}e^{-t/\tau_{2}}+I_{0}$. The fit yields the characteristic decay times $\tau_{1}^{K}$ = 5.8 ± 2.3 ps and $\tau_{2}^{K}$ = 58.7 ± 9.3 ps. The power‐law, *I*∝*t* ^−β^, utilized to account for the pump‐probe data at long delay times, yields the power‐law exponent β^*K*^ = 0.37 ± 0.01. Table S2 (Supporting Information) summarizes the fit parameters obtained for NaPHI and CsPHI.

We assign the τ_1_ to the rapid decay of photoexcited excitons, either by recombination or population of shallow traps. τ_2_ is associated with the decay of the shallow traps, giving rise to the population of deeply trapped electrons. These two ultrafast components account for roughly 58% decay of the initial ESA amplitude of KPHI. This value is similar for aqueous NaPHI (55%), but for CsPHI, 69% of the initially observed TA amplitude decays already in the first 100 ps. Such biexponential decay behavior with exciton recombination and trap‐assisted charge recombination has been previously observed in aqueous K,Na‐PHI.^[^ ^12^ ^]^ Monitoring of non‐emissive trapped states and the exponential tail of trap states below band edges related to recombination via charge trapping/de‐trapping in conventional PCNs has also been reported.^[^ ^21^ ^]^ The fraction of excitations in the sub‐100 ps time regime that end up in deep traps is almost identical for KPHI (0.33 ± 0.04) and NaPHI (0.34 ± 0.03), however, slightly fewer excitations appear to end up in deep traps in CsPHI (0.27 ± 0.01). These values refer to the biexponential fit of the TA data recorded up to a delay time of 100 ps, and refer to the non‐decaying component in this fit (see the factor *I*_0_ in Table S2, Supporting Information).

For NaPHI and CsPHI, the kinetic analysis yields $\tau_{1}^{Na}$ = 4.1 ± 0.6 ps, $\tau_{2}^{Na}$ = 54.7 ± 8.3 ps and $\tau_{1}^{Cs}$ = 1.1 ± 0.6 ps, $\tau_{2}^{Cs}$ = 45.9 ± 1.8 ps, respectively. This indicates that the larger Cs ions modulate the properties of the trap states in the material to an extent that the shallow traps are destabilized and the rate of their population increases about fivefold in comparison to that of KPHI. Upon increasing the cation size from NaPHI to KPHI, τ_1_ and τ_2_ increase, while τ_1_ and τ_2_ decrease when moving from KPHI to CsPHI. Considering the amplitudes associated with the time constants, A_1_ decreases and A_2_ increases with increasing cation size from NaPHI to CsPHI (Table S2, Supporting Information). This behavior cannot be rationalized solely based on the modulation of trap state density as inferred from the emission measurements, and its molecular origin remains to be studied in detail.

The TA decay at delay times larger than 100 ps reflects the charge recombination from deep traps.^[^ ^6^, ^20^, ^21^, ^22^ ^]^ A quantitative analysis of the respective TA kinetics identifies the power‐law exponent β to be 0.38 ± 0.01 for all PHIs investigated here. However, the TA signal for all three PHIs does not completely decay within the measurement time window, and the presence of a residual long‐lived signal reflects the persistence of long‐lived photogenerated charges in the materials. These charges are likely trapped in deep traps, associated with surface functional groups.^[^ ^21^ ^]^ Therefore, the relaxation decay kinetics of the charge carriers from initially shallow to deep traps probably occur in longer times, from few‐100 ns to µs range.

### 2.5 Transient Absorption in the Presence of a Hole Quencher

In a second step, photoexcited Na‐, K‐, and CsPHI were investigated in the presence of EtOH as a hole scavenger. EtOH was added to an aqueous solution of the PHIs (concentration of 1 g L^−1^) in concentrations varying from 5 to 30 vol.%. As mentioned before, the addition of 10 vol.% EtOH to KPHI decreases the TA signal intensity without causing spectral changes in the broad ESA band centred ≈665 nm (Figure 4b). The accelerated decay of the ESA features of KPHI (Figure 5b) reflects a shortening of the characteristic time constant $\tau_{1}^{K}$ and $\tau_{2}^{K}$ upon the addition of EtOH (Figure 5d). Drawing on our previous assignment of $\tau_{1}^{K}$ and $\tau_{2}^{K}$, we conclude that EtOH quenches holes, thereby opening an additional decay pathway for near‐band edge excitons and holes from shallow traps. Also $A_{1}^{K}$, the amplitude associated with the decay of near‐band edge excitons, increases in the presence of EtOH. As fewer holes relax into shallow traps, the amplitude reflecting the decay of the latter species, $A_{2}^{K}$, decreases in the presence of EtOH (Figure 5c,d). However, the sum of $A_{1}^{K}$ and $A_{2}^{K}$ remains constant upon varying the EtOH. Moreover, $I_{0}^{K}$, the signal amplitude remaining after 100 ps, remains almost unaffected by EtOH within the range of 0.33–0.40, i.e., the number of excitations ending up in deep trapped states is insensitive to the presence of EtOH. This points to the fact that different subsets of excitonic excitations exist in the material, which either decay via excitonic and shallow‐trap mediated recombination within 100 ps or directly via deep‐trap assisted recombination beyond 100 ps. Considering the power‐law fit of the long‐delay time TA data, the EtOH concentration invariance of β^*K*^ = 0.32 ± 0.04 suggests that the deep‐trap assisted recombination is unaffected by EtOH. In other words, while shallow trapped charges are prone to be quenched by EtOH, the deeply trapped photogenerated charges on ns timescales do not react with EtOH. The deep traps can be ascribed to electronic states enabled by the presence of edge groups.^[^ ^6f^ ^]^ While the spectroscopic signatures of electrons and holes are spectrally congested in PHIs, it is inferred from our data that, in the presence of EtOH, electrons and not holes primarily populate the deep traps, a process which is chemically equivalent to the formation of PHI^●–^ radical anions.^[^ ^6f^ ^]^

**Figure 6:**
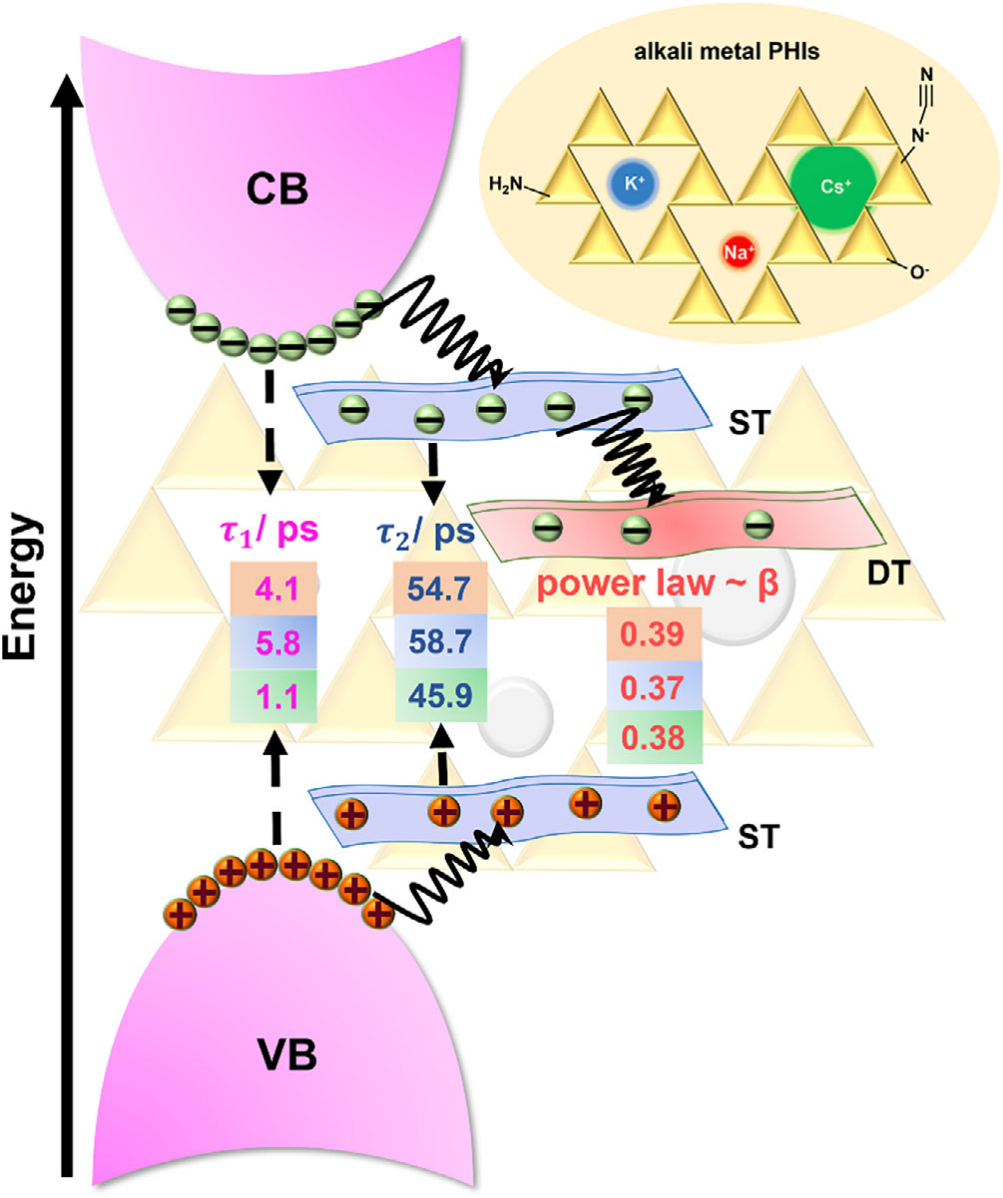
Schematic representation of the proposed charge carrier transport model in the three water‐soluble alkali metal PHIs – NaPHI, KPHI, and CsPHI. The numbers in pink refer to the corresponding characteristic time constant (τ_1_) associated with the decay of excitons, the numbers in blue are correlated to the second characteristic time constant (τ_2_) as a result of the decay of shallow traps within the 100 ps time regime, and the β values in red are attributed to the power‐law exponent beyond 100 ps. τ_1_, τ_2_ and β, highlighted with light red, light blue, and light red represent these time constants for Na‐, K‐, and CsPHI, respectively. CB represents the conduction band, VB represents the valence band, ST and DT are denoted by shallow trap and deep trap, respectively.

Figure 6 depicts a schematic summary of the model inferred from the TA data for PHIs. The model suggests the decay kinetics of PHIs to be a combination of decay of primary excitons with a characteristic time constant τ_1_ and the decay of shallow‐trapped excitons with a time constant τ_2_. Notably, when comparing the different PHIs, the longer the characteristic lifetimes τ_1_ and τ_2_, the higher the photocatalytic activity, i.e., CsPHI << NaPHI < KPHI (compare Figures 6 and 1b) can be observed. The presence of EtOH leads to effective quenching of the photogenerated holes in PHIs, ultimately oxidizing EtOH to acetaldehyde. While the data recorded for NaPHI in the presence of EtOH (Figure S11a and Table S3, Supporting Information) matches the results obtained for KPHI, CsPHI behaves differently under otherwise identical conditions (Figure S11b, Supporting Information). Most notably, the decay kinetics of CsPHI in the presence of 5 and 10 vol.% EtOH show no significant changes as compared to that of CsPHI without the addition of EtOH (see Table S3 in Supporting Information for the results of the data fitting). We ascribe this to the large size of the Cs ion, which exfoliates the individual PHI layers and hence decreases the density of the trap states available to be quenched by low concentrations of EtOH. Furthermore, no clear trend in the TA decay kinetics is visible upon analysing the characteristic time constants and amplitudes of CsPHI in the presence of EtOH of different concentrations. These findings are significant as they strongly suggest that the inferior activity of CsPHI in photocatalytic H_2_O_2_ production, as compared to KPHI and NaPHI (see Figure 1b), is directly related to less effective extraction of holes from CsPHI by electron donors. In this context, two points are noteworthy. First, photocatalytic H_2_O_2_ production at PHIs requires the presence of alcohols as effective electron donors and was negligible in the absence of ethanol. Second, it should be mentioned that an extensive TA spectroscopic investigation of all our PHI materials in the presence of additional (apart from dissolved O_2_) *electron* quencher (AgNO_3_) revealed only insignificant effects on TA decay kinetics at relatively low concentrations of AgNO_3_ (< 0.4 mM), whereby the more pronounced effects observed at higher concentrations are likely to be caused by undesired effects due to increased ionic strength and/or formation of Ag nanoparticles (for comprehensive discussion see Note S1, Figures S12, S13, and Table S4, Supporting Information).

### 2.6 Understanding the Effect of Various Cations on Exciton Dynamics in PHIs

Our spectroscopic data (vide supra) provide several valuable insights that represent an excellent phenomenological basis for understanding the different activities of water‐soluble KHI, NaPHI, and CsPHI in photocatalytic H_2_O_2_ production (Figure 1b). First, steady‐state and time‐resolved (ns timescale) emission spectroscopy revealed the presence of two distinct types of emissive states, likely associated with surface and/or defect‐bound excitons. Notably, the analysis of excitation wavelength dependence and the characteristic half‐lifetimes of emission decays pointed to the significant effect of specific cations on the overall trap distribution in the PHIs. For example, the data suggest a lower concentration of surface defect‐related excitonic states in NaPHI, which is in line with the relatively larger particle size of NaPHI as compared to KPHI and CsPHI (see Figure 1e) and the lower propensity of the small Na^+^ cation to exfoliate the layered PHI. Second, ultrafast (fs timescale) TAS provided two‐fold crucial information on the excitonic dynamics and its direct impact on photocatalytic activity: i) characteristic time constants associated with the decay of primary excitons (τ_1_) and excitons trapped in shallow traps (τ_2_) are, in a remarkable way, fully in line with the photocatalytic activity increasing in the order CsPHI << NaPHI < KPHI (Figures 6 and 1b); ii) an analysis of the differences in excitonic dynamics obtained in the presence and absence of EtOH as hole scavenger clearly indicated that the inferior photocatalytic activity of CsPHI, as compared to KPHI and NaPHI, is related to less effective hole extraction by EtOH. In this context, it should be noted that the TAS and emission experiments probe fundamentally different subsets of excited states, which accounts for the observed discrepancies between their kinetics. Emission measurements are sensitive predominantly to radiative recombination of bright excitons and emissive trap states, typically representing only a small fraction of the overall excited‐state population due to their low radiative efficiency, characteristic of disordered carbon nitrides and heterogeneous semiconductors. This is corroborated by very low values of relative photoluminescence quantum yields (PLQY) at 370 nm excitation of 0.7% for KPHI, 1.2% for NaPHI, and 1.3% for CsPHI. Thus, emissive (bright) states constitute only a minor fraction of photoexcited carriers. TAS measurements, in contrast, detect both emissive and non‐emissive states, including those critically involved in charge separation and photocatalysis. In other words, the vast majority of photogenerated excitations in our materials relax into non‐emissive states, which dominate TAS signals and drive catalytic processes.

Excitingly, the above‐mentioned phenomenological insights from spectroscopy can be directly linked to our recent theoretical work on the relation between the structure and exciton dynamics in PCNs.^[^ ^13^ ^]^ Notably, we proposed the strong influence of dark (i.e., selection rule‐forbidden, here specifically momentum‐forbidden) excitons on the photoactivity of various PCNs since – in contrast to bright excitons – the formation and the lifetimes of the dark excitons were more significantly affected by variations of the microstructure of PCNs, in particular by varying interactions between heptazine units. In structural terms, we found that dark exciton lifetimes can be significantly extended depending on the degree of interlayer interaction and corrugation, primarily due to lone‐pair (LP) electron interactions. Specifically, two primary mechanisms responsible for dark exciton lifetimes in carbon nitrides were identified. The first mechanism involving long‐lived (deep‐trapped) excitons, which result from forbidden transitions caused by minimal LP–LP orbital overlap due to pronounced corrugation, was demonstrated for graphitic (fully condensed) domains in conventional (*non‐ionic*) carbon nitrides. The second mechanism, proposed to be prominent in *ionic* carbon nitrides (PHIs), involves swift interlayer exciton transfers enabled by enhanced *π*–*π* orbital interactions arising from optimized interlayer stacking pattern, and is associated with shallow‐trapped dark excitonic states. These findings are further supported by the observation that strong exciton binding energies (> 2 eV) computed for various monolayer microstructural models are substantially reduced to < 1 eV upon stacking, indicating that microstructural features and interlayer interactions are paramount factors governing the excitonic effects in PCN derivatives, including PHIs.

**Figure 7:**
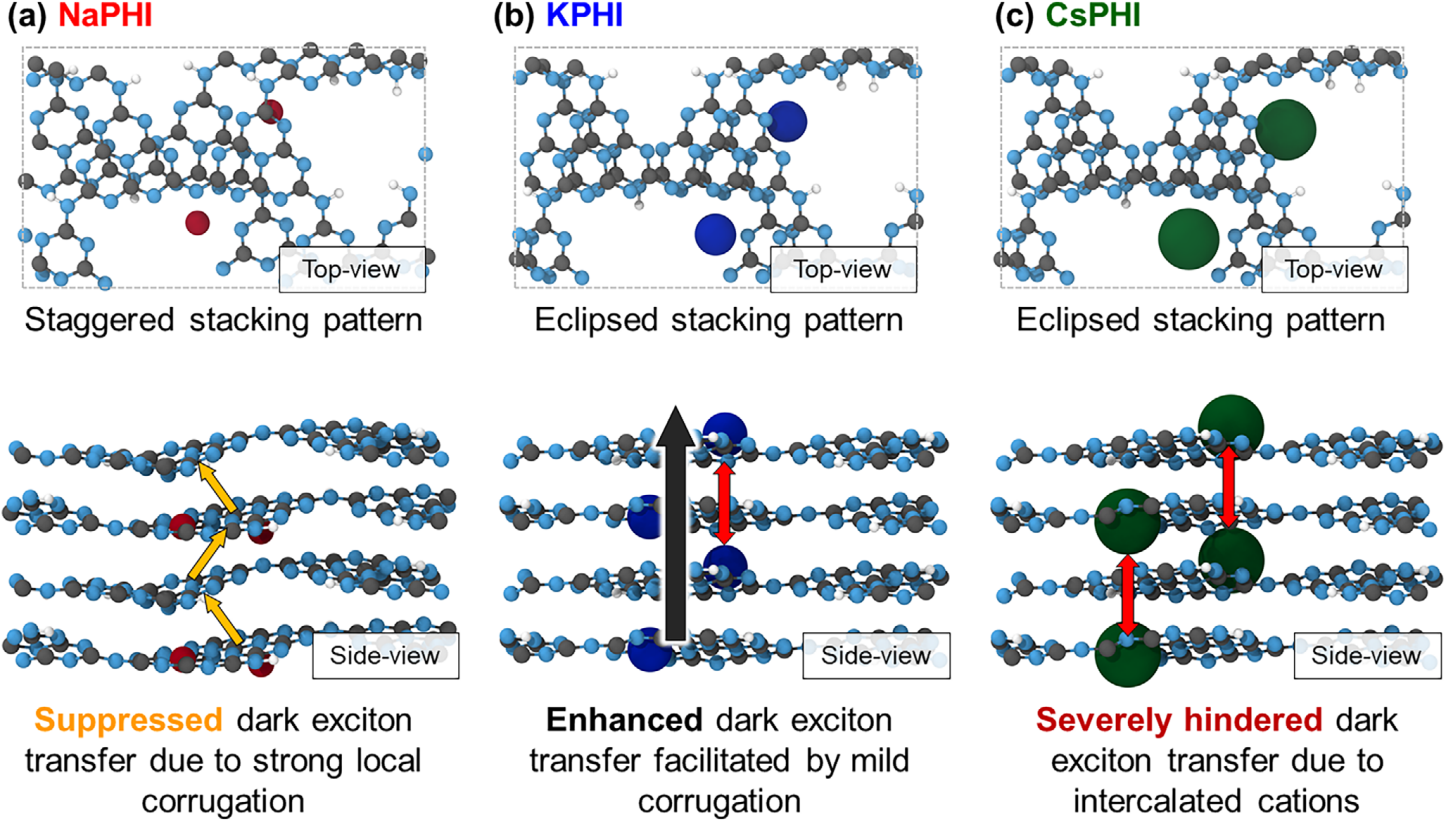
Schematic illustration of the proposed dark exciton transfer dynamics in PHIs as a function of the intercalated cation species: a) NaPHI, b) KPHI, and c) CsPHI.

Drawing on these results, we now provide a detailed theoretical analysis of the influence of specific cations on the PHI structures, particularly with respect to the feasibility of interlayer dark exciton transfer and related enhancement of exciton lifetimes. First, the changes in the electronic structure of KPHI, NaPHI, and CsPHI induced by varying cation contents were evaluated by calculating the projected densities of states (PDOS) (Figures S14 and S15, Supporting Information). Nevertheless, the band‐edge contributions of the cations remained subtle, so the effects of their content and positions were negligible. This made it challenging to directly explain the variations in the behavior of different materials. On the other hand, it unequivocally suggests that cations do not affect the electronic transitions and excitonic effects *directly*, but rather *indirectly*, *i.e*., due to the PHI structural changes induced by their presence. Importantly, the systematic analysis of Bader charges (Figures S16–S19, Supporting Information) and changes in effective band centers (Figure S20 and Tables S5 and S6, Supporting Information) provides critical insights into the influence of different cations and their interactions on the PHI structure and corresponding excitonic effects (**Figure** **7**). In NaPHI (Figure 7a), Na ions primarily interact with nitrogen LP electrons, leading to corrugation‐related exciton decay patterns typical for PHIs.^[^ ^13a^ ^]^ Due to its small ionic radius, Na^+^ induces stronger localized corrugation when intercalated between the layers, as compared to KPHI and CsPHI or to (hypothetical) cation‐free PHI structure. Since Na^+^ induces significant corrugation within the layers, it has a slightly detrimental effect on the overall interlayer interactions typical in PHIs, limiting the exciton lifetimes (Figure S21, Supporting Information). In contrast, KPHI exhibits interactions that vary depending on the positioning of K^+^ ions, which engage either with LP electrons or π electrons in the PHI structure. The less pronounced corrugation in KPHI, as compared to NaPHI, enhances these characteristics, resulting in a more versatile interlayer exciton dynamics as compared to NaPHI (Figure 7b). Finally, the analysis of CsPHI predicts a distinct behavior due to the much larger ionic radius of Cs^+^ cation, which makes it prone to being positioned between the layers. However, the firm positioning of Cs^+^ between layers not only induces enhanced structural disorder (see the XRD data in Figure 1c), but also restricts significantly the PHI interlayer interactions (Figure 7c). This suppression of interlayer interactions, particularly those involving phonon‐derived vibrational modes,^[^ ^23^ ^]^ affects the *π*–*π* orbital electron interactions critical for effective interlayer exciton transfer to the surface, where it can react with an electron donor.

To summarize, our theoretical framework provides a basis to interpret the observed modulation of exciton lifetimes as being primarily related to different dark exciton dynamics governed by changes in interlayer interactions due to the altered structural corrugation in the presence of various cations. While Na^+^ affects the interlayer interactions minimally, K^+^ exerts a partial beneficial effect on them, and Cs^+^ heavily suppresses them. The interlayer dark exciton transfer is predicted to be highly beneficial for effective scavenging of holes by electron donor (such as EtOH) at the PHI surface, which is a key process in avoiding recombination and establishing photocatalytic turnover. Thus, our results establish a consistent rational link between the observed photocatalytic activity (Figure 1b), the exciton lifetimes (Figure 6), and structural features of *ionic* carbon nitrides with different cations. Moreover, they suggest that the superior photocatalytic performance of PHI materials employing mixed cations (e.g., Na^+^ and K^+^) reported in the literature^[^ ^3b^ ^]^ might be understood in terms of more delicate structural tuning that renders the interlayer dark exciton dynamics beneficial. More broadly, our results provide additional evidence for the critical role of dark exciton dynamics in photocatalysis, in line with the recent findings of Zhao et al. on anatase TiO_2_.^[^ ^24^ ^]^

## 3 Conclusion

In this work, we have, for the first time, established a direct and consistent correlation between photocatalytic activity, detailed spectroscopic characterization, and theoretical analysis of structural effects on exciton dynamics in an important class of materials – *ionic* (PHI‐type) polymeric carbon nitrides. Through a combination of steady‐state and time‐resolved emission spectroscopy, ultrafast transient absorption measurements, and comprehensive theoretical modeling, we demonstrate that the nature of intercalated cations exerts a profound indirect influence on dark exciton dynamics by modulating the structural corrugation and interlayer interactions within PHI frameworks. Our spectroscopic results reveal systematic trends in exciton lifetimes and trapping behavior, which align remarkably well with the photocatalytic performance of water‐soluble ionic carbon nitrides with different cations (KPHI, NaPHI, and CsPHI) in H_2_O_2_ production. In particular, ultrafast transient absorption spectroscopy resolved two excitonic relaxation pathways – a rapid (sub‐100 ps) decay via excitonic and shallow traps and a slower (sub‐nanosecond) decay via deep traps. Notably, only the exciton and shallow‐trap populations were sensitive to hole scavenging by ethanol, whereby the effect was more pronounced for shallow‐trapped species. As a result, the markedly inferior H_2_O_2_ generation by CsPHI can be attributed to its inefficient hole extraction, evidenced by the negligible change in its exciton and shallow‐trap decay kinetics upon ethanol addition, in contrast to the significant acceleration of decay processes observed for NaPHI and KPHI in the presence of ethanol. These experimental insights are further supported by theoretical analysis, which elucidates how specific cation‐framework interactions control the structure of PHIs and govern the feasibility of interlayer dark exciton transfer – a key factor in determining photocatalytic activity. Notably, we show that Na⁺ maintains the characteristic PHI exciton behavior with minimal disruption to interlayer dynamics, K⁺ partially enhances interlayer exciton transfer, while Cs⁺ severely hampers these processes due to structural disorder and restricted interlayer coupling. This study thus provides a comprehensive and rational framework linking material structure, exciton dynamics, and photocatalytic function in PHIs. Beyond advancing the fundamental understanding of excitonic processes in polymeric carbon nitrides, our findings offer valuable design principles for tailoring photocatalytically active PHI‐based materials through controlled tuning of dark exciton dynamics by modification of interlayer interactions.

## 4 Experimental Section

### Materials

Water‐soluble, aqueous alkali metal cation poly (heptazine imide) (PHIs) with different concentrations (NaPHI, KPHI, and CsPHI with concentrations of 1.1, 1.2, and 1.6 g L^−1^, respectively) were prepared following Krivtsov et al. and diluted to proper concentrations according to the experimental requirement.^[^ ^3b^ ^]^ The synthesis procedure involves thermal condensation of melamine with different metal hydroxide melts (NaOH, KOH, or CsOH) at 330 °C to yield surface functionalized heptazine‐based NaPHI, KPHI, and CsPHI, respectively. For details of the synthesis and characterization methods, see the Supporting Information. Ethanol (EtOH) (pure, 200 proof) was obtained from Sigma–Aldrich and used without further purification. Silver nitrate (AgNO_3_) was obtained from Sigma–Aldrich, and 3.4 mg of AgNO_3_ was dissolved in 10 mL of water to prepare a 2 mM stock solution.

### Photocatalytic H_2_O_2_ Evolution

The photocatalytic activity of the PHI materials in the production of H_2_O_2_ was investigated over a period of 4 h. To this end, a PHI solution of 20 mL with a PHI concentration of 0.75 g L^−^1 containing 10 vol.% ethanol (VWR, 99.8%) was prepared and irradiated by a UV LED (365 nm, photon flux of 50 mW cm^−2^). The temperature was kept constant at 22 °C using a thermostat (Julabo 200F). During the experiment, the solution was stirred under air. The H_2_O_2_ concentration was determined photometrically; after 0, 1, 2, and 4 h, samples (0.5 mL) were taken and 0.5 mL TiOSO_4_ solution (Ti 1 wt.%) in sulfuric acid was added. The addition of the TiOSO_4_ caused the catalyst to coalesce, so that it could be easily removed using a 0.2 µm PTFE syringe filter. The filtered solution was analysed using a Cary 60 (Agilent Technologies) UV–vis spectrophotometer. The absorbance values at 420 nm were taken to estimate the concentration of the produced H_2_O_2_. Beforehand, a calibration curve was constructed using standard H_2_O_2_ solutions, following the same procedure as described before. The control experiments in the absence of any photocatalyst showed no observable formation of H_2_O_2_. Standard errors were calculated from at least three experiments, and error bars represent the 95% confidence interval.

### Steady‐State UV–vis Absorption and Emission Spectroscopy

The steady‐state absorption spectra were measured on a Jasco‐V780 UV‐vis‐NIR spectrophotometer using 1 cm quartz cuvettes. Water‐soluble PHIs were irradiated using a 365 nm LED (Thorlabs, M365LP1) with the irradiance of 75 mW mm^−2^ within the UV–vis spectrometer for time‐lapse UV–vis experiments. The UV–vis spectra under illumination at 365 nm were collected after 10 min of irradiation, followed by 2 min in the dark for 12 cycles. Steady‐state emission spectra were recorded using an FLS980 emission spectrometer (Edinburgh Instruments Ltd, the United Kingdom) equipped with an ozone‐free Xenon arc lamp (450 W) as an excitation source. The measurements were performed at various excitation wavelengths from 340 to 520 nm using 1 cm quartz cuvettes with a spectral resolution of Δλ_ex._ = 1 nm and Δλ_em._ = 2 nm, in the excitation and emission channels, respectively. Optical density with varying excitation wavelengths and different PHIs varies between 0.1 and 0.0002. PHIs with a concentration of 1 g L^−1^ were prepared from the respective PHI stock solutions to measure steady‐state absorption, and also in the presence of 10 vol.% EtOH and 0.2 mM AgNO_3_. Steady‐state emission measurements were conducted on the samples with the same optical density, i.e., 0.1, at 340 nm.

### Time‐Resolved Emission Spectroscopy

Spectrally resolved emission decays were measured using a Hamamatsu streak scope C4334 (Hamamatsu Photonics, Japan) in photon counting mode with a time window of 1.7 ns. The sample was excited with a frequency‐doubled output of a Ti‐sapphire laser (Tsunami, Newport Spectra‐Physics GmbH, the USA) at 370 nm at a pulse repetition rate of 80 MHz. The instrument response function was measured using the SiO_2_ nanoparticles. The emissions from the sample were collected by a Chromex 250IS 3 imaging spectrograph.

### Time‐Resolved Transient Absorption Spectroscopy

Femtosecond transient absorption measurements were performed using a home‐built experimental setup at room temperature in 1 mm pathlength quartz cuvettes.^[^ ^25^ ^]^ A white‐light supercontinuum probe pulse at 1 kHz repetition rate was generated by focusing a minor part of the output of the amplified Ti:Sapphire laser system (Libra, Coherent) into a rotating CaF_2_ crystal. After splitting the white light into a probe and a reference, the probe pulse was focused onto the sample using a concave mirror with a focal length of 500 nm. The spectra of probe and reference were detected by a Czerny‐Turner spectrograph (SP2150, Princeton Instruments, 150 mm focal length) equipped with a diode array detector (Pascher Instruments AB, Sweden). The 325 nm 100‐fs pump pulses were generated by a TOPAS‐White. The average pump power was set to 120 mW, corresponding to an estimated fluence of ≈96 µJ cm^−2^. The repetition rate of the pump pulses was reduced to 0.5 kHz by a mechanical chopper, and the mutual polarization between the pump and probe was adjusted to the magic angle (54.7°). Having passed the sample, the pump pulses were blocked and the probe intensity was measured as a function of the optical delay (within a time range of 10 ns) between the probe and pump pulses. For the data analysis, the data were first chirp‐corrected using coherent artifact signals,^[^ ^26^ ^]^ and subsequently a sum of exponential functions was fitted to the data using a global fitting routine.

### Statistical Analysis

Statistical methods, including data presentation and sample sizes, were provided in the corresponding figure and table captions.

## Conflict of Interest

The authors declare no conflict of interest.

## Supporting information



Supporting Information

## Data Availability

The data that support the findings of this study are openly available in Zenodo at https://doi.org/10.5281/zenodo.15462475, reference number 15462475.
