# Supplementary material for: Photocatalytic Activity of Ionic Carbon Nitrides is Governed by Cation‐Modulated Dark Exciton Dynamics
Source: Adv Sci (Weinh). 2025 Aug 21;12(42):e09312. doi: 10.1002/advs.202509312 (PMC12622463; doi:10.1002/advs.202509312)
Supplement: Supplementary file 1 — Supporting Information [file ADVS-12-e09312-s001.docx]

Supporting Information

**Photocatalytic Activity of Ionic Carbon Nitrides is Governed by Cation-Modulated Dark Exciton Dynamics**

Arindam Konar,^‡a,b^ Johannes Liessem,^‡c^ Changbin Im,^c^ Mohamed M. Elnagar,^c^ Dariusz Mitoraj,^c^ Pratibha Saini,^a^ Igor Krivtsov,^d^ Sarah Jasmin Finkelmeyer,^b^ Jan Griebel,^j^ Martin Presselt,^b,e,f^ Timo Jacob,^c,g,h*^ Radim Beranek,^c*^ Benjamin Dietzek-Ivanšić^a,b,j*^

^a^ Institute for Physical Chemistry, Friedrich Schiller University Jena, Lessingstrasse 4, 07743 Jena, Germany

^b^ Leibniz Institute of Photonic Technology (Leibniz-IPHT), Research Department Functional Interfaces, Albert-Einstein-Strasse 9, 07745 Jena, Germany

^c^ Institute of Electrochemistry, Ulm University, Albert-Einstein-Allee 47, 89081 Ulm, Germany

^d^ Department of Chemical and Environmental Engineering, University of Oviedo, 33006 Oviedo, Spain

^e^ Center for Energy and Environmental Chemistry Jena (CEEC Jena), Friedrich Schiller University Jena, Philosophenweg 7a, 07743 Jena, Germany

^f^ Sciclus GmbH & Co. KG, Moritz-von-Rohr-Str. 1a, 07745 Jena, Germany

^g^ Helmholtz-Institute-Ulm (HIU) Electrochemical Energy Storage, 89081 Ulm, Germany

^h^ Karlsruhe Institute of Technology (KIT), 76021 Karlsruhe, Germany

^j^ Leibniz Institute of Surface Engineering (IOM), Permoserstraße 15, 04318 Leipzig, Germany

*Corresponding authors: [timo.jacob@uni-ulm.de](mailto:timo.jacob@uni-ulm.de), [radim.beranek@uni-ulm.de](mailto:radim.beranek@uni-ulm.de), [benjamin.dietzek@uni-jena.de](mailto:benjamin.dietzek@uni-jena.de)

^‡^These authors contributed equally to this work.

**Synthesis of water-soluble PHI photocatalysts**

For the syntheses of the photocatalysts, melamine (99%, Sigma Aldrich), NaOH (99%, Merck), KOH (99%, Sigma-Aldrich) and CsOH$\cdot$*x*H_2_O (99%, Thermo Scientific, water content 15-20%) were used.

The alkali metal hydroxides were dried at 120 °C for 2 hours. Afterwards, the corresponding poly(heptazine imide) (PHI) was prepared by quickly mixing and grinding 1.5 g of melamine with appropriate quantities of the alkali metal hydroxide (see **Table** **S1**). The fine powder was heated up in a muffle furnace (Carbolite) to 330 °C in a lid-covered crucible at a rate of 5 °C min^-1^. Then the temperature was kept for 2 h at the reached temperature. The obtained solid was ground and mixed with 100-150 mL of deionized water. The insoluble part was removed by centrifugation, followed by filtration, first by a paper filter and then by a 0.2 μm PTFE syringe filter. The solution of the water-soluble alkali metal PHI nanoparticles was put inside a cellulose membrane with a pore size of 3.5 kDa, and dialyzed against deionized water until the pH of the water phase was below 8. Eventually, the concentration of the stock solution was determined gravimetrically, and adjusted to a concentration above 0.9 g L^-1^ by evaporation at 45 °C. **Table S1** summarizes the synthetic conditions applied for the preparation of the photocatalysts, and the figure below shows the typical appearance of water-soluble PHIs at a concentration of 0.75 g L^–1^.

**Table S1. Synthetic parameters for the preparation of Na-, K-, and CsPHI**

| Sample name | NaOH [mmol] | KOH [mmol] | CsOH [mmol] | Melamine [mmol] | XRD phase composition |
| --- | --- | --- | --- | --- | --- |
| NaPHI | 15 |  |  | 12 | PHI |
| KPHI |  | 15 |  | 12 | PHI |
| CsPHI |  |  | 15 | 12 | PHI |

**
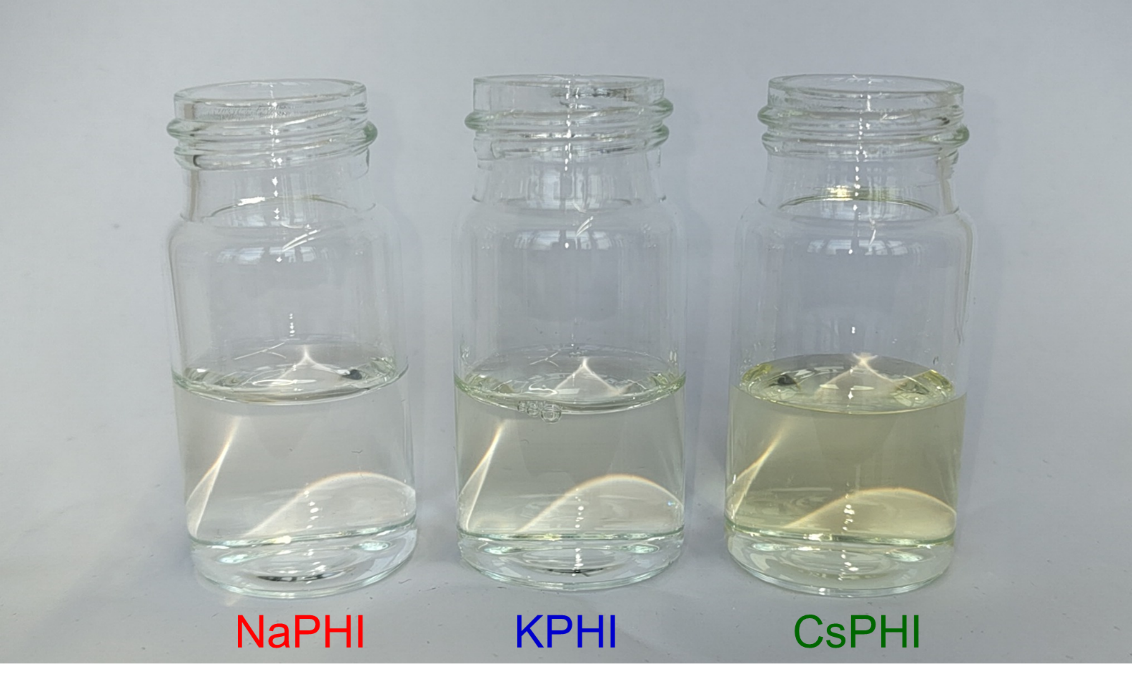
**

**Preparation of the films from the PHI solution using the drop-casting method**

The films from the water-soluble PHI photocatalysts were prepared via a droop-casting technique. Initially, the catalysts were dissolved in ultrapure water to create a 1 mg mL^-1^ aqueous solution. For the fabrication of the films, a measured volume of the prepared solution was carefully drop-cast onto thoroughly cleaned quartz substrates, aiming for uniform coverage across the entire substrate surface to ensure reproducibility and consistent optical measurements. After the deposition, the coated substrates were dried in a laboratory oven at 60 ℃ overnight.

**Energy Dispersive X-ray Spectroscopy (EDS) and elemental mapping**

EDS and elemental mapping analyses of the PHI materials were performed using a ZEISS LEO 1550 VP scanning electron microscope (SEM) operated at 15 kV, equipped with an energy-dispersive X-ray spectroscopy (EDS) system (Ametek, USA). For sample preparation, 0.5 mL of each PHI dispersion was drop-cast onto titanium foil (used as a conductive substrate) and air-dried. The chemical composition and elemental distribution were subsequently examined.

**Electron Paramagnetic Resonance (EPR) Spectroscopy**

EPR measurements were made at room temperature with X-band EPR spectrometer ELEXSYS (BRUKER) equipped with a TE102 cavity. The microwave power was set to 20 mW and the modulation amplitude to 1 G. The spectra were analysed with the software WinEPR. The light source was a Omnicure LX500 UV lamp with a 365 nm LED head.

**Relative photoluminesce quantum yield (PLQY) measurements**

The relative PLQY measurement were measured at 370 nm excitation with quinine sulfate in 0.5 M H_2_SO_4_ as a reference dye with a known PLQY value of 0.546. The relative PLQY values were calculated using the equation


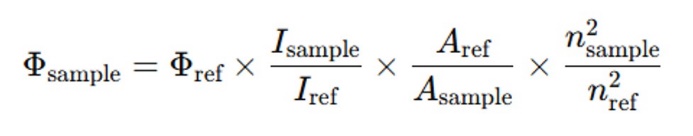


where Φ_sample_**​** is PLQY of the sample, Φ_ref_ is PLQY of the reference, *I*_sample_​ and *I*_ref_ are the fluorescence emission intensities of the **sample and reference, respectively,** *A*_sample_​ and *A*_ref_ are the absorbances at the excitation wavelength of the **sample and reference, respectively, and** *n*_sample_​ and *n*_ref_ are the refractive indexes of the sample and refernce solutions, respectively.

**X-ray diffraction (XRD) analysis**

To record the x-ray diffractogram (Malvern Panalytical, X´Pert MPD Pro, Cu-source, 2 h) of the materials, the water was evaporated completely at 70 °C and ground to a fine powder.

**Fourier-transform infrared (FTIR) spectroscopy**

The spectra were recorded using a Shimadzu IRTracer-100 spectrometer at a resolution of 4 cm^–1^ from the samples dried, ground and pressed into KBr pellets.

**Photothermal deflection spectroscopy (PDS)**

The photothermal deflection spectroscopy (PDS) setup used here consists of a light source (LOT-QD; 1000 W Xe high pressure lamp and a 300 mm monochromator (LOT-QD MSH 300) optimized for a maximum intensity of 200 nm to 2500 nm). The light is modulated by a chopper (Thorlabs) with a frequency of 5 Hz and focused on a spot size of 2 × 6 mm on the sample through a f = 75 mm lens. The intensity of the incident light (250 nm: 2.9-6.5 µW, 265 nm: 9.9-12.7 µW, 275 nm: 14.7 µW, 410 nm: 90 µW, 1100 nm: 174 mW) is monitored using a quartz glass plate as a beam splitter placed between the focusing lens and the sample, and a trans-impedance amplified silicon detector (Thorlabs). The deflection of a 0.5 mW HeNe laser (LINOS, λ = 633 nm, HeNe 633-0.8-PO, beam diameter of 0.49 mm) is measured with a lateral effect sensor (Thorlabs PDA90). The deflection and reference signals were read out using two lock-in amplifiers (Stanford Research Systems SRS-830). The whole system is controlled by a self-written Labview program, which collects all data and also corrects the PDS signal according to the incident light intensity. A glassy carbon plate (3 mm × 30 mm) was used as a reference sample. The samples - thin films on ITO glass - were fixed in the center of a quartz glass cuvette (CV10Q3500F, Thorlabs) filled with FC-40 (Sigma Aldrich). The PDS measurement was carried out from 200 nm to 2500 nm in 5 nm steps. With the monochromator slits set to 4 mm, the spectral resolution was 10 nm in the wavelength range of 200-1000 nm and 20 nm in the wavelength range of 1000-1600 nm. For selected samples, higher-resolution PDS measurement was conducted in 2.5 nm steps. In this case, the monochromator slits were adjusted to 2 mm, yielding a spectral resolution of 5 nm in the 200-1000 nm range and 10 nm in the1000-1600 nm range.

**UV-vis diffuse reflectance spectroscopy (DRS)**

UV-vis diffuse reflectance spectroscopy (DRS) measurements of the prepared films out of the PHI solutions were performed using a Jasco V-780 UV-vis-NIR spectrophotometer equipped with a thin film holder.

**Dynamic Light Scattering (DLS) and Zeta Potential measurements**

All measurements were carried out using the native water-soluble PHI solutions after dialysis at pH~8. Particle size measurements were performed with a Zetasizer Pro from Malvern Panalytical by Dynamic Light Scatttering (DLS)-Non-invasive Back Scatter (NIBS) using 900-1000 μL of sample in a Folded Capillary Zeta Cell (DTS1080) with a measurement angle of 173°, in a diameter range of 0.3 nm-10 μm at 22 °C. Every size measurement was performed multiple times. The size distribution and zeta potential were analyzed 1-3 days after completion of the dialysis.

Zeta potential measurements were also performed with a Zetasizer Pro from Malvern Panalytical by Mixed-Mode Measurement phase analysis light scattering (M3-PALS) using a disposable folded capillary cell (DTS1080) in a diameter range of 0.3 nm-10 μm. Every zeta potential measurement was performed five times, and error bars represent the 95% confidence interval (mean ± *t* × SE; SE = standard error; *t* = critical value of the *t*-distribution).

**Computational details**

All calculations were carried out using version 6.4.0 of the Vienna Ab initio Simulation Package (VASP), which utilizes the projector-augmented wave (PAW) method.^[1]^ The plane wave basis set was truncated with an energy cut-off of 400 eV, and the wavefunctions were optimized to a convergence criterion of 10^−6^ eV. Atomic positions were relaxed until the residual forces were below 5 × 10−2 eV ˚A^-1^. Gaussian smearing with a width of 0.01 eV was employed to account for finite-temperature effects, and long-range interactions were treated using D3 dispersion corrections.^[2]^ Structural relaxations were performed with the Perdew-Burke-Ernzerhof (PBE) exchange-correlation functional within the generalized gradient approximation (GGA).^[3]^ For Brillouin zone integration, a Γ-centered 2 × 2 × 2 k-point grid was used. To achieve higher accuracy, pseudopotentials were modified by including partial valence electrons for Na and K, effectively treating some core electrons as valence electrons. This approach enhances the accuracy for metallic or complex compounds. Additionally, for Cs, pseudopotentials were generated by treating semi-core electrons as valence electrons, which is particularly advantageous for d-block elements or systems requiring high precision.


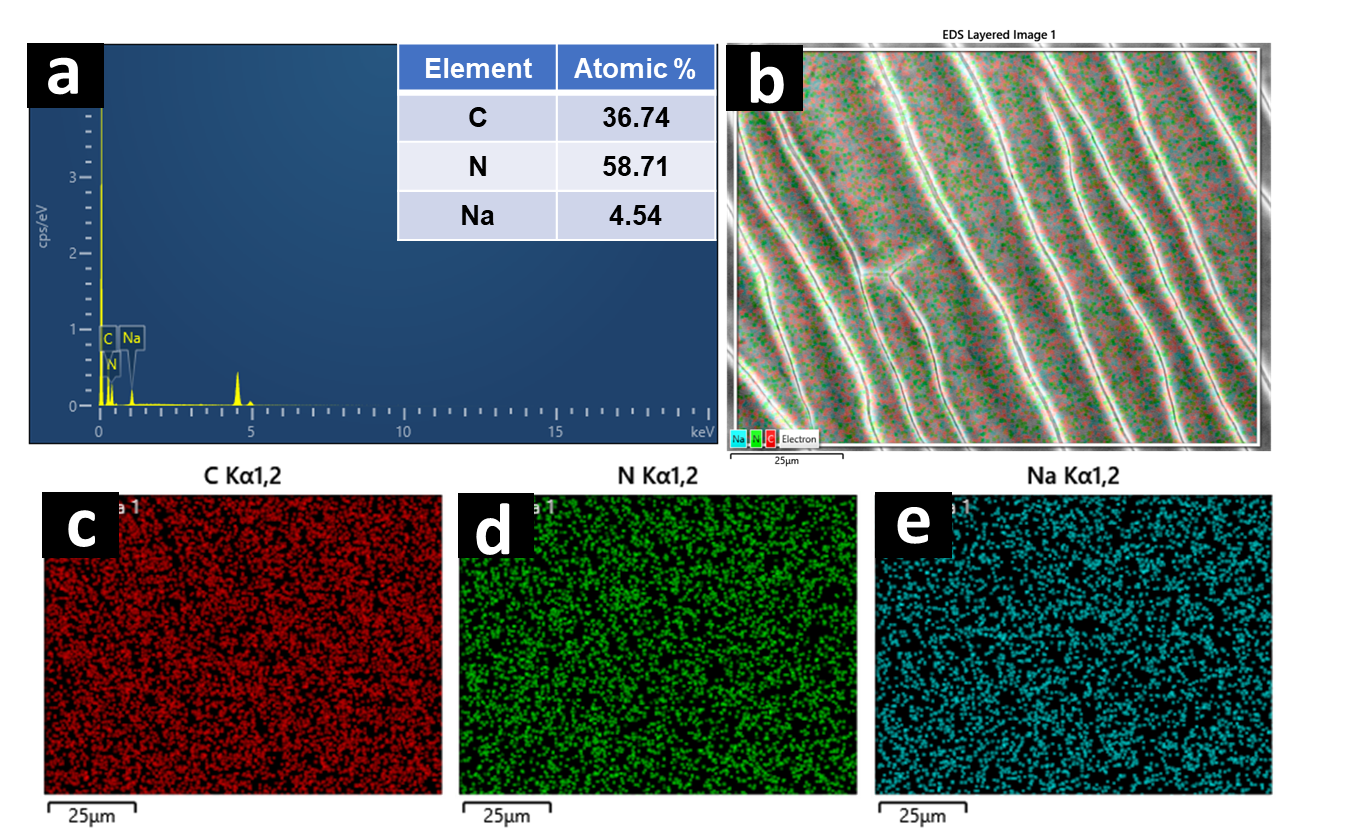


**Figure S1.** (a) A representative EDS spectrum of NaPHI. (b) Elemental mapping overlay image showing the combined spatial distribution of C, N, and Na. Individual elemental distributions are shown for (c) carbon (C), (d) nitrogen (N), and (e) sodium (Na). The elemental mapping was performed from three different areas, and atomic percentage of Na content was 4.7±0.1% (mean ± standard error calculated from three measurements from different areas).


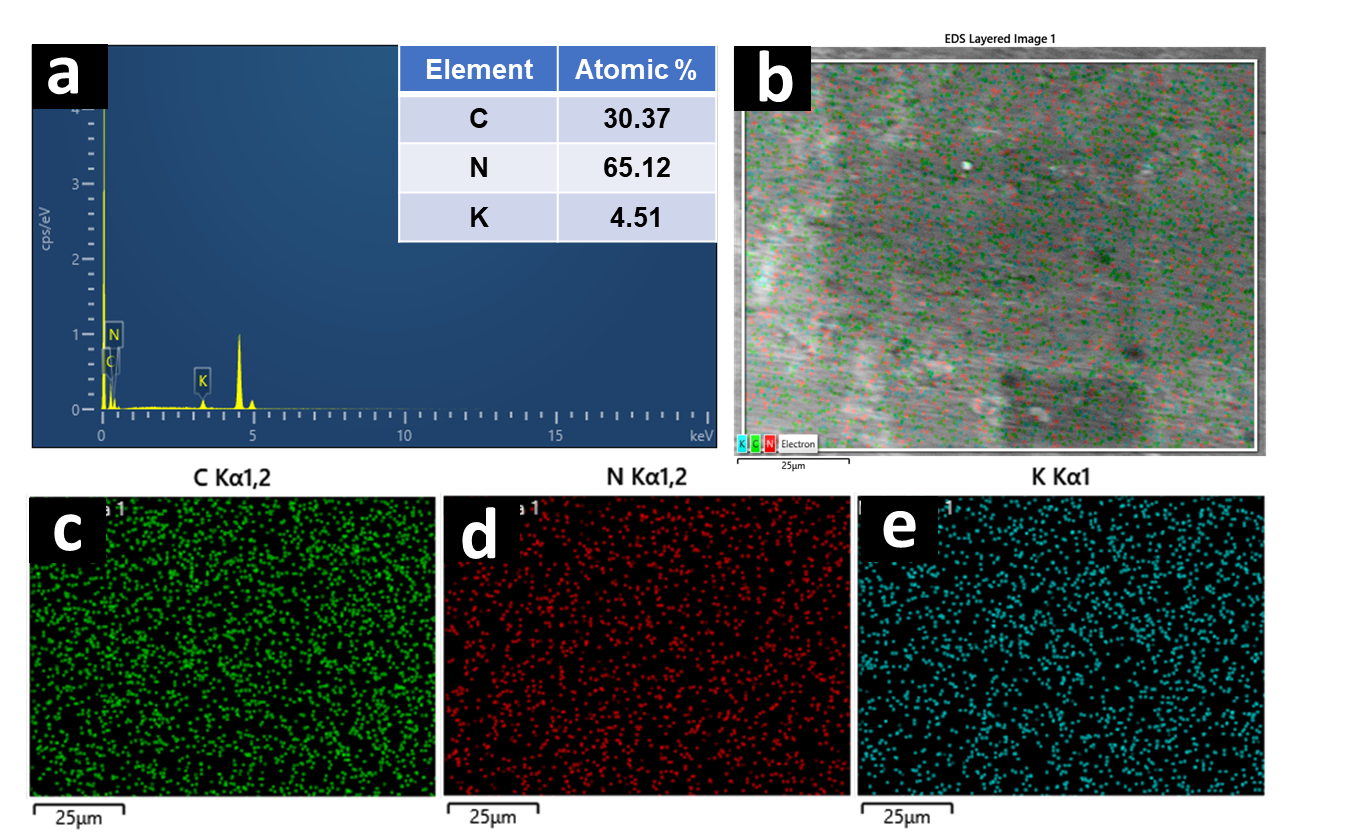


**Figure S2.** (a) EDS spectrum of KPHI. (b) Elemental mapping overlay image showing the combined spatial distribution of C, N, and K. Individual elemental distributions are shown for (c) carbon (C), (d) nitrogen (N), and (e) potassium (K). The elemental mapping was performed from three different areas, and atomic percentage of K content was 4.4±0.2% (mean ± standard error calculated from three measurements from different areas).


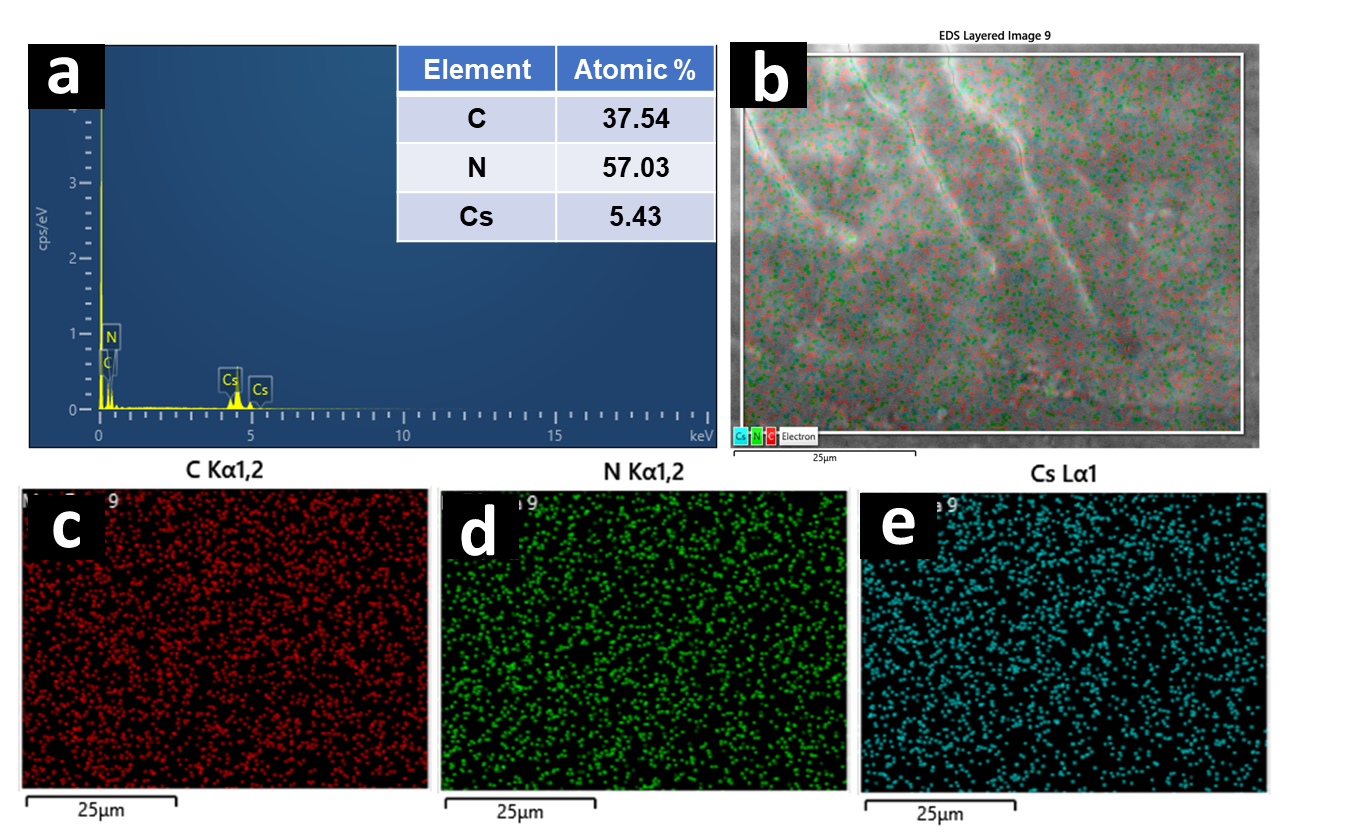


**Figure S3.** (a) EDS spectrum of CsPHI. (b) Elemental mapping overlay image showing the combined spatial distribution of C, N, and Cs. Individual elemental distributions are shown for (c) carbon (C), (d) nitrogen (N), and (e) cesium (Cs). The elemental mapping was performed from three different areas, and atomic percentage of Na content was 5.5±0.8% (mean ± standard error calculated from three measurements from different areas).


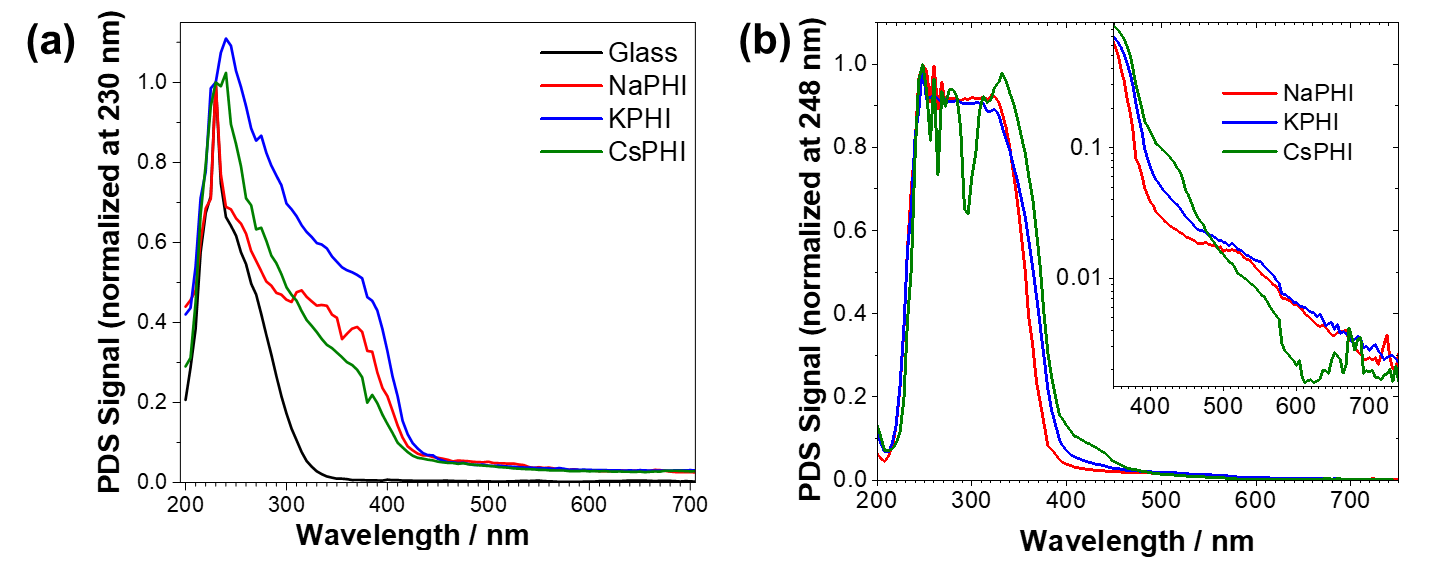


**Figure S4.** Photothermal deflection spectra (PDS) recorded at (a) thin (~400-500 nm) films on glass obtained from water-soluble PHI samples using gelation and calcination, and at (b) thicker drop-casted PHI films.


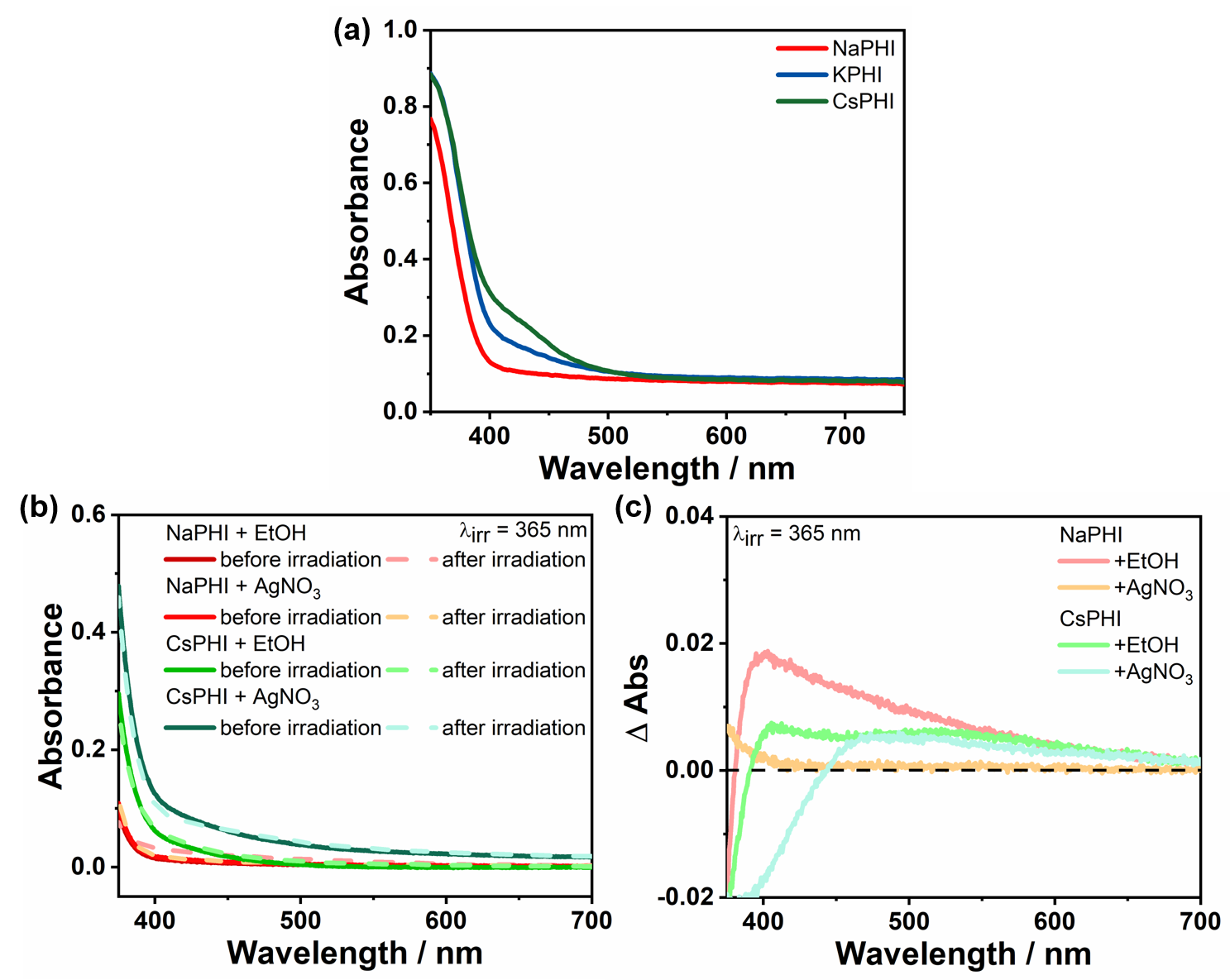


**Figure S5.** (a) UV-vis diffuse reflectance spectra (DRS) from drop-casted PHI films. The y-axis is labelled as ‘absorbance’ based on the empirical relationship $A={-log}_{10} (R)$, where (R) is the measured reflectance. This approximation is commonly used to visualize optical absorption trends and mimics the behavior of absorbance in transmission-based measurements, even though no transmission occurs in DRS. (b) UV-vis absorption spectra of NaPHI and CsPHI (concentration of 1 g L^‒1^) in the presence of 10 vol% EtOH and 0.2 mM AgNO_3_ before (solid lines) and after (dashed lines) 2 hours of 365 nm LED irradiation. (c) Differential absorption spectra of NaPHI and CsPHI under the same illumination conditions in the presence of EtOH (10 vol%) and AgNO_3_ (0.2 mM).


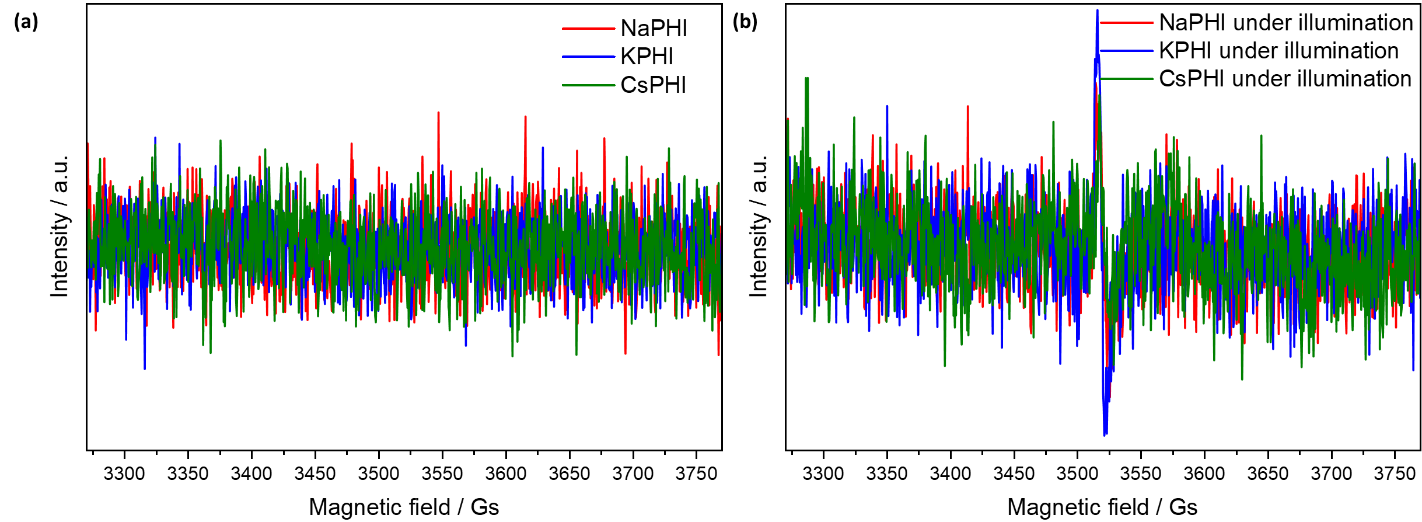


**Figure S6**. EPR spectroscopy of NaPHI, KPHI and CsPHI in the presence of ~ 10 %Vol EtOH under inert atmosphere in the dark **(a)** and under light irradiation with 365 nm **(b)**. Under irradiation, the formation of PHI^●–^ is evidenced by a broad signal of low intensity at ~3519 G, corresponding to the g-factor of 2.0019. This value is in line with g-factors reported for PHI^●–^ anion radicals in the literature.^[4,5]^ Note: Due to strong microwave absorption by water, EPR measurements required concentrating the water-soluble PHI solutions to the highest practical levels, resulting in increased viscosity and line broadening. Concentrations of PHIs used here therefore differ from those in the rest of the study.


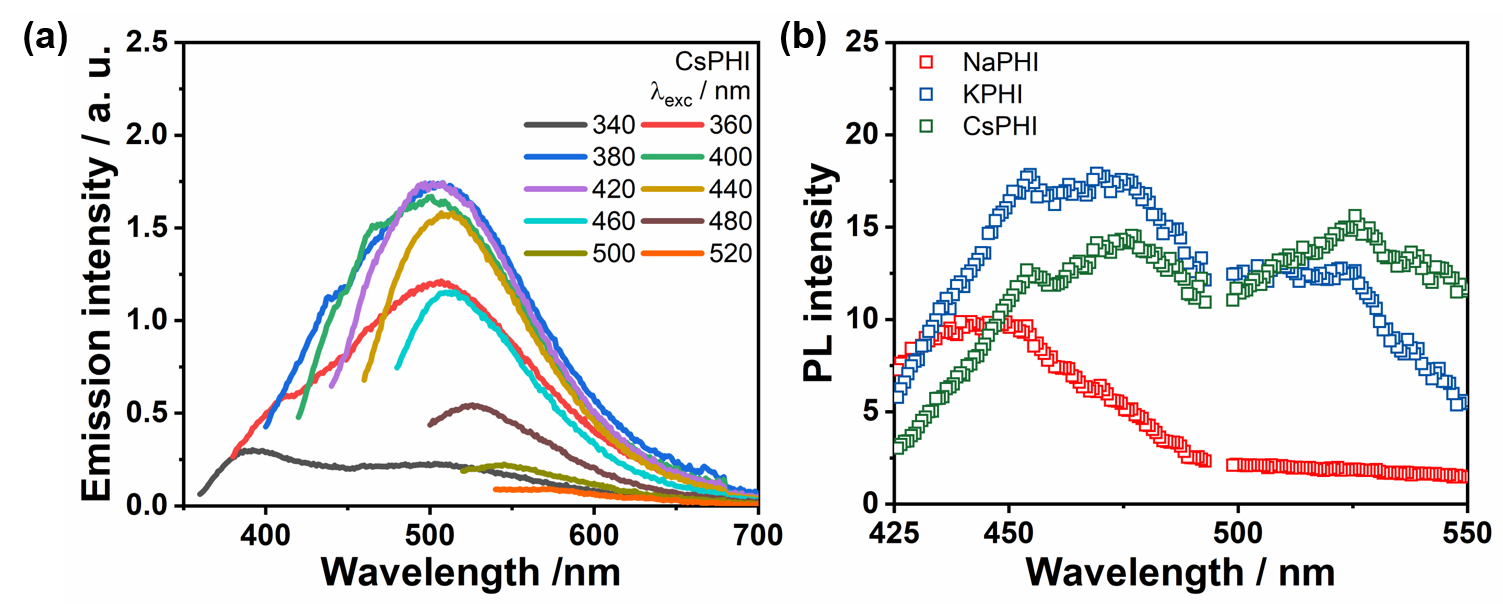


**Figure S7.** (a) Steady-state emission spectra of CsPHI (concentration 1 g L^‒1^) with various excitation wavelengths. Optical densities at excitation wavelengths vary from 0.1 (λ_exc_ = 340 nm) to 0.0002 (λ_exc_ = 520 nm). (b) Photoluminescence spectra (obtained by integrating the signal obtained from time-resolved emission measurements over the measurement time window) of Na-, K-, and CsPHI upon excitation at 370 nm.


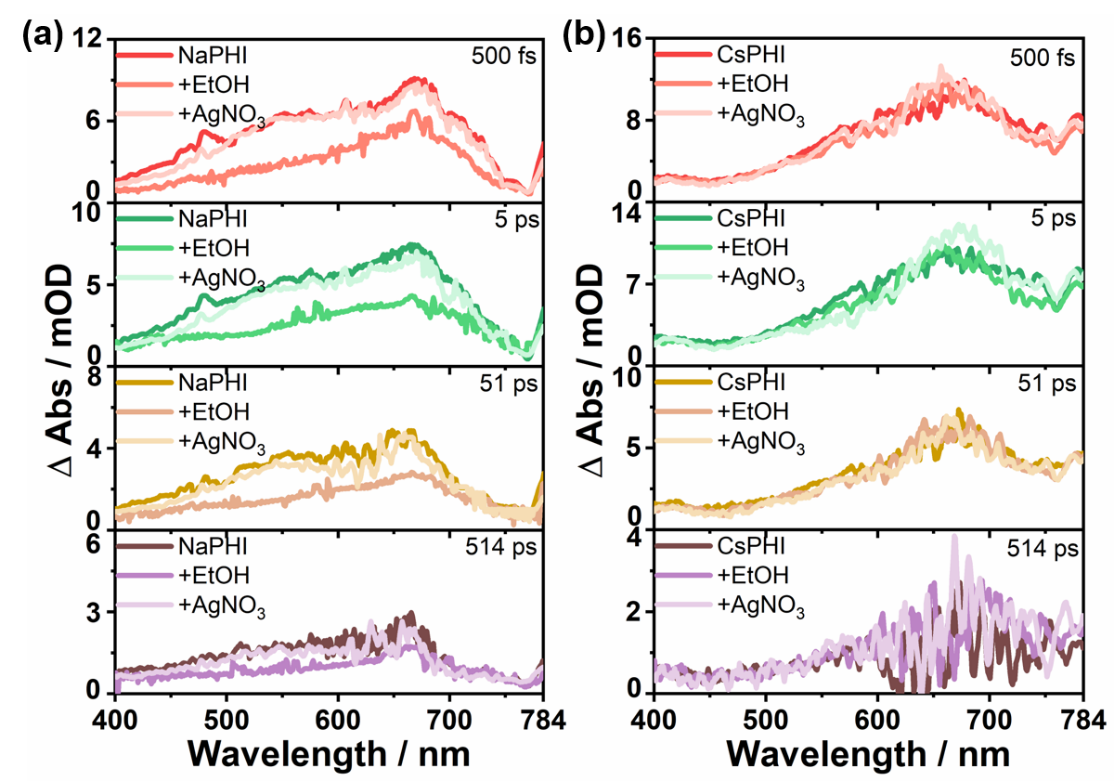


**Figure S8.** fs-transient absorption spectra of (a) NaPHI and (b) CsPHI with concentration of 1 g L^‒-1^ at selected time delays in the presence of 10 vol% EtOH and 0.2 mM AgNO_3_ upon excitation at 325 nm.


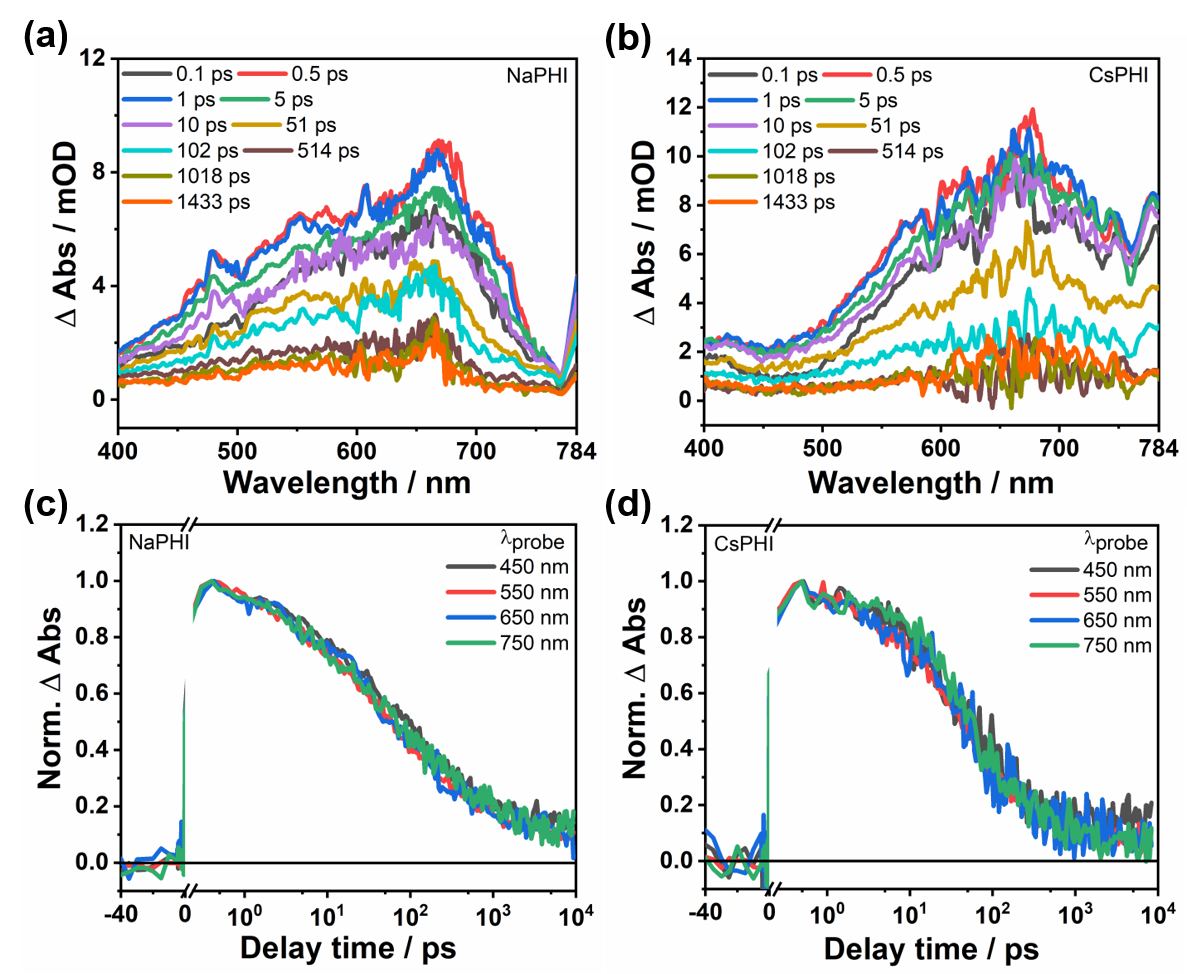


**Figure S9.** fs-transient absorption spectra of (a) NaPHI (concentration 1 g L^‒1^) and (b) CsPHI (concentration 1 g L^‒1^) measured at selected time delays after excitation at 325 nm. Normalized fs-transient absorption decay kinetics of (c) NaPHI (concentration 1 g L^‒1^) and (d) CsPHI (concentration 1 g L^‒1^) at selected wavelengths of 450, 550, 650 and 750 nm.


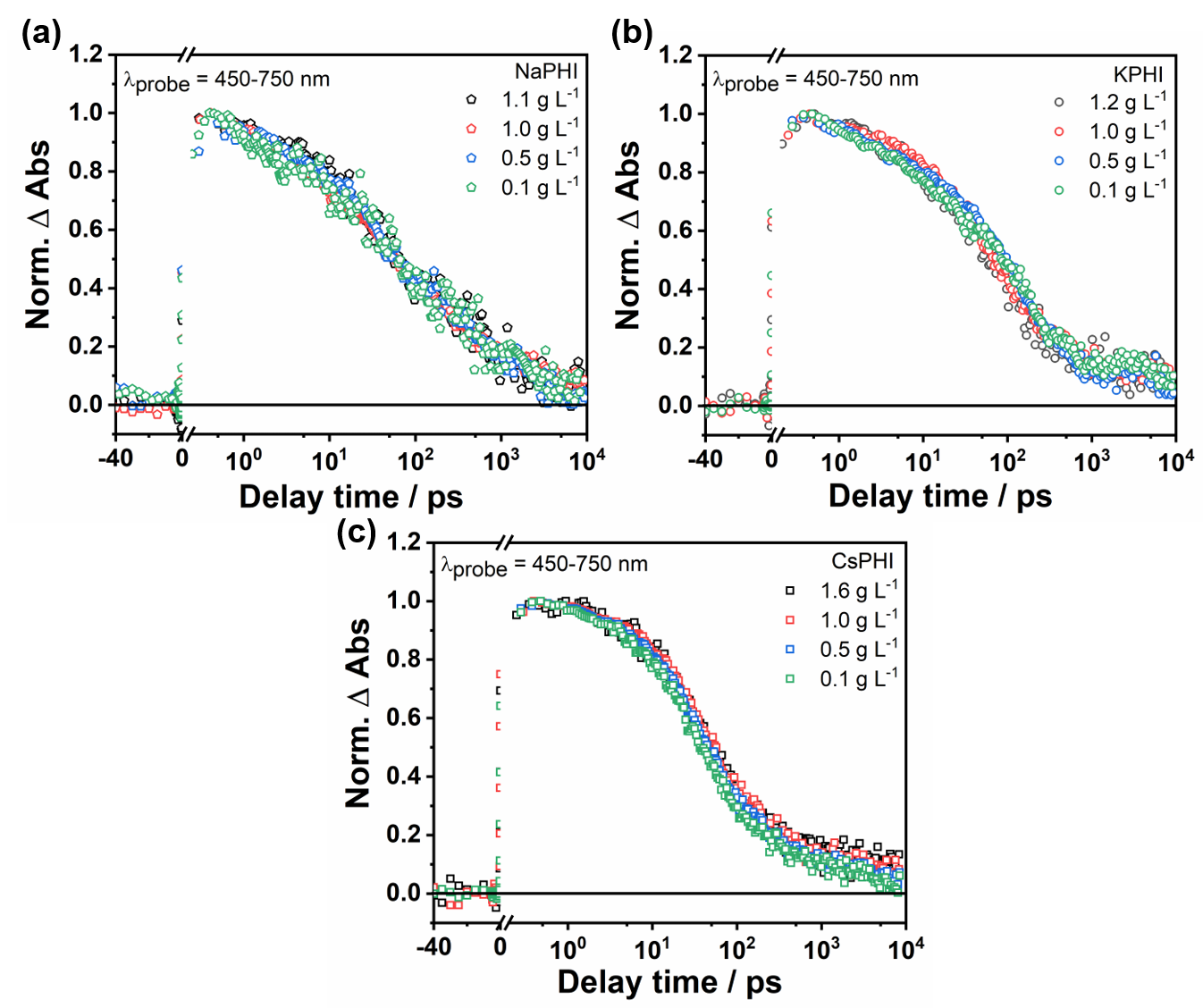


**Figure S10.** Normalized fs-transient absorption decay traces of (a) NaPHI, (b) KPHI and (c) CsPHI excited at 325 nm at various concentrations.


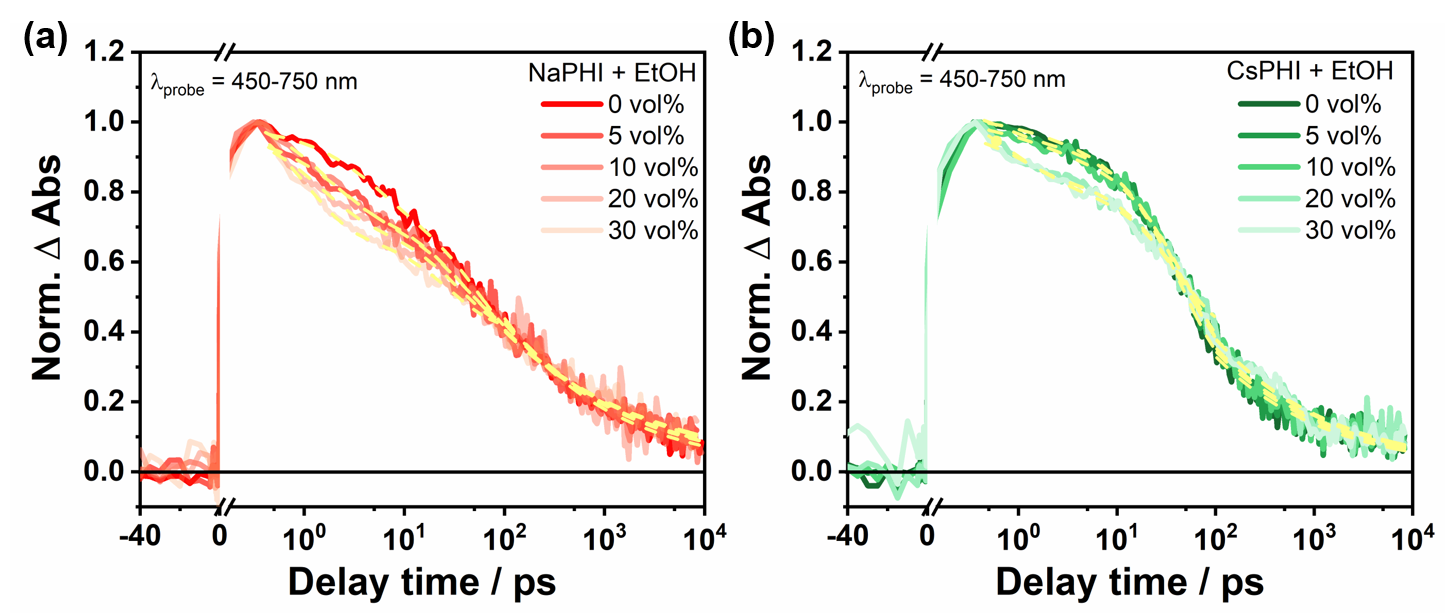


**Figure S11.** Normalized fs-transient absorption decay kinetics of (a) NaPHI and (b) CsPHI dispersed in water-ethanol mixtures at various volume ratios (0 to 30 vol%).

**Table S2.** Values of the fit parameters for the fs-transient absorption decay kinetics of NaPHI, KPHI and CsPHI, each having a concentration of 1 g L^–1^. The TA kinetics of all the PHIs can be biexponentially fitted within the first 100 ps by $I=A_{1}e^{-t/\tau_{1}}+A_{2}e^{-t/\tau_{2}}+I_{0}$ and at the longer delay times, the decay kinetics are represented by a power-law relationship: $I\propto t^{-\beta}$, where I denotes the TA signal at a specific probe wavelength at a specific delay time, $\tau_{1}$ and $\tau_{2}$ are two characteristic time constants, $A_{1}$ and $A_{2}$ are the respective amplitudes associated with $\tau_{1}$ and $\tau_{2}$, $I_{0}$ represents the approximation for the fraction of excitations within 100 ps that end up in deep traps, and β is the exponent of the power-law. Each sample was measured in triplicate, and the errors are given as *±σ* (*σ* is the standard deviation of the fitted parameter obtained from the nonlinear regression using OriginLab software)*.*

| Fit parameters | PHIs | | |
| --- | --- | --- | --- |
|  | NaPHI | KPHI | CsPHI |
| $I_{0}$ | 0.34 ± 0.03 | 0.33 ± 0.04 | 0.27 ± 0.01 |
| $A_{1}$ | 0.19 ± 0.01 | 0.07 ± 0.02 | 0.04 ± 0.02 |
| $\tau_{1}$ | 4.1 ± 0.6 | 5.8 ± 2.3 | 1.1 ± 0.6 |
| $A_{2}$ | 0.46 ± 0.01 | 0.56 ± 0.02 | 0.71 ± 0.01 |
| $\tau_{2}$ | 54.7 ± 8.3 | 58.7 ± 9.3 | 45.9 ± 1.8 |
| β | 0.39 ± 0.01 | 0.37 ± 0.01 | 0.38 ± 0.02 |

**Table S3.** Fit parameter values for the fs-transient absorption decay kinetics of NaPHI (top), KPHI (middle) and CsPHI (bottom) (concentration of 1 g L^–1^) with different ethanol concentrations ranging from 0 to 30 vol%. The TA kinetics of all the PHIs can be biexponentially fitted within the first 100 ps by $I=A_{1}e^{-t/\tau_{1}}+A_{2}e^{-t/\tau_{2}}+I_{0}$ and at the longer delay times, the decay kinetics are represented by a power-law relationship: $I\propto t^{-\beta}$, where I denotes the TA signal at a specific probe wavelength at a specific delay time, $\tau_{1}$ and $\tau_{2}$ are two characteristic time constants, $A_{1}$ and $A_{2}$ are the respective amplitudes associated with $\tau_{1}$ and $\tau_{2}$, $I_{0}$ represents the approximation for the fraction of excitations within 100 ps that end up in deep traps, and β is the exponent of the power law. Each sample was measured in triplicate, and the errors are given as *±σ* (*σ* is the standard deviation of the fitted parameter obtained from the nonlinear regression using OriginLab software)*.*

| Fit parameters | NaPHI with different EtOH concentrations | | | | |
| --- | --- | --- | --- | --- | --- |
|  | 0 vol% | 5 vol% | 10 vol% | 20 vol% | 30 vol% |
| $I_{0}$ | 0.34 ± 0.03 | 0.39 ± 0.03 | 0.40 ± 0.02 | 0.40 ± 0.01 | 0.41 ± 0.01 |
| $A_{1}$ | 0.19 ± 0.01 | 0.22 ± 0.02 | 0.27 ± 0.02 | 0.26 ± 0.02 | 0.37 ± 0.02 |
| $\tau_{1}$ | 4.1 ± 0.6 | 2.1 ± 0.5 | 1.5 ± 0.2 | 1.4 ± 0.2 | 1.0 ± 0.1 |
| $A_{2}$ | 0.46 ± 0.01 | 0.37 ± 0.02 | 0.37 ± 0.01 | 0.33 ± 0.01 | 0.29 ± 0.01 |
| $\tau_{2}$ | 54.7 ± 8.3 | 41.2 ± 7.9 | 38.8 ± 4.2 | 35.4 ± 4.2 | 29.4 ± 2.3 |
| β | 0.39 ± 0.01 | 0.31 ± 0.01 | 0.35 ± 0.01 | 0.35 ± 0.02 | 0.30 ± 0.01 |

| Fit parameters | KPHI with different EtOH concentrations | | | | |
| --- | --- | --- | --- | --- | --- |
|  | 0 vol% | 5 vol% | 10 vol% | 20 vol% | 30 vol% |
| $I_{0}$ | 0.33 ± 0.04 | 0.34 ± 0.02 | 0.36 ± 0.02 | 0.40 ± 0.01 | 0.40 ± 0.02 |
| $A_{1}$ | 0.07 ± 0.02 | 0.12 ± 0.02 | 0.24 ± 0.01 | 0.24 ± 0.01 | 0.32 ± 0.04 |
| $\tau_{1}$ | 5.8 ± 2.3 | 2.0 ± 0.5 | 2.0 ± 0.2 | 1.5 ± 0.1 | 1.4 ± 0.3 |
| $A_{2}$ | 0.56 ± 0.02 | 0.49 ± 0.02 | 0.39 ± 0.01 | 0.34 ± 0.01 | 0.32 ± 0.02 |
| $\tau_{2}$ | 58.7 ± 9.3 | 47.1 ± 5.1 | 46.3 ± 4.9 | 31.2 ± 1.8 | 23.8 ± 4.1 |
| β | 0.37 ± 0.01 | 0.40 ± 0.02 | 0.34 ± 0.02 | 0.34 ± 0.02 | 0.35 ± 0.02 |

| Fit parameters | CsPHI with different EtOH concentrations | | | | |
| --- | --- | --- | --- | --- | --- |
|  | 0 vol% | 5 vol% | 10 vol% | 20 vol% | 30 vol% |
| $I_{0}$ | 0.27 ± 0.01 | 0.31 ± 0.02 | 0.30 ± 0.02 | 0.40 ± 0.02 | 0.27 ± 0.03 |
| $A_{1}$ | 0.04 ± 0.02 | 0.11 ± 0.06 | 0.07 ± 0.02 | 0.21 ± 0.05 | 0.16 ± 0.02 |
| $\tau_{1}$ | 1.1 ± 0.6 | 0.6 ± 0.3 | 1.3 ± 0.6 | 0.7 ± 0.2 | 1.3 ± 0.3 |
| $A_{2}$ | 0.71 ± 0.01 | 0.65 ± 0.02 | 0.63 ± 0.02 | 0.46 ± 0.01 | 0.56 ± 0.03 |
| $\tau_{2}$ | 45.9 ± 1.8 | 46.9 ± 2.7 | 52.3 ± 3.9 | 41.2 ± 3.2 | 62.6 ± 6.7 |
| β | 0.38 ± 0.02 | 0.37 ± 0.02 | 0.37 ± 0.01 | 0.36 ± 0.02 | 0.38 ± 0.01 |

***Note 1: Transient absorption spectroscopy in the presence of AgNO_3_ as an electron quencher.***

Furthermore, we investigated the impact of the electron scavenger AgNO_3_ on the photoinduced dynamics in KPHI (**Figure S8a**). As mentioned before, upon addition of AgNO_3_ in low concentration ($\leq$0.2 mM), the TA signals appear unaltered (**Figure 4b**). Upon increasing the AgNO_3_ concentration to 0.4 mM, a very minute change in the kinetics can be observed, as a consequence of which the time-constants reflecting the sub-100 ps data are altered, *i.e.*, a decrease in $\tau_{1}^{K}$ from 4.9 to 2.3 ps and a slight increase in $\tau_{2}^{K}$ from 56.7 to 64.6 ps are noted compared to the time constants in the absence of AgNO_3_. The corresponding amplitudes $A_{1}^{K}$ and $A_{2}^{K}$ remain constant upon increasing AgNO_3_ concentration up to 0.4 mM. Also, the decay kinetics at longer time scale (> 100 ps), as represented by the power-law remains unaltered, and so is $\beta^{K}$, which assumes a value of 0.35 ± 0.01 in the presence of 0.4 mM AgNO_3_ (**Figure S8b** and **Table S4**). However, further increasing the AgNO_3_ concentration from 0.6 to 1 mM leads to an acceleration of the TA decay. On longer timescales, *i.e.* ~200 ps, the addition of Ag^+^ causes a steeper decrease of the TA kinetics (**Figure S8a**), which indicates an onset of electron quenching when larger amounts of AgNO_3_ are added to the sample. Upon further increasing the AgNO_3_ concentration up to 1 mM, the excited state absorption decay speeds up significantly, reflecting the fact that the electrons become effectively quenched. A very negligible change in $\tau_{1}^{K}$, decreasing from 1.0 to 0.6 ps, associated with the exciton decay, is observed upon increasing AgNO_3_ concentration from 0.6 to 1 mM. A similar observation can be noticed for $\tau_{2}^{K}$, which results in a slight decrease from 32.7 to 22.8 ps with increasing AgNO_3_ concentration from 0.6 to 1 mM. This suggests that higher AgNO_3_ concentration induces additional decay pathways *via* electron quenching and therefore results in a depopulation of shallow trapped electrons, causing a decrease in $\tau_{2}^{K}$. In contrast, the power-law exponent $\beta^{K}$ remains in the range of 0.22 ± 0.03 upon increasing AgNO_3_ concentration from 0.6 mM to 1 mM. Thereby, $\beta^{K}$ tends to decrease with increasing AgNO_3_ concentrations, and a clear difference in the range of $\beta^{K}$ from 0.36 ± 0.02 in the presence oflow concentrations of AgNO_3_ ($\leq$ 0.4 mM) to 0.22 ± 0.03 with higher AgNO_3_ concentrations is evident when comparing the deep-trap assisted decay kinetics of KPHI with different AgNO_3_ concentrations.


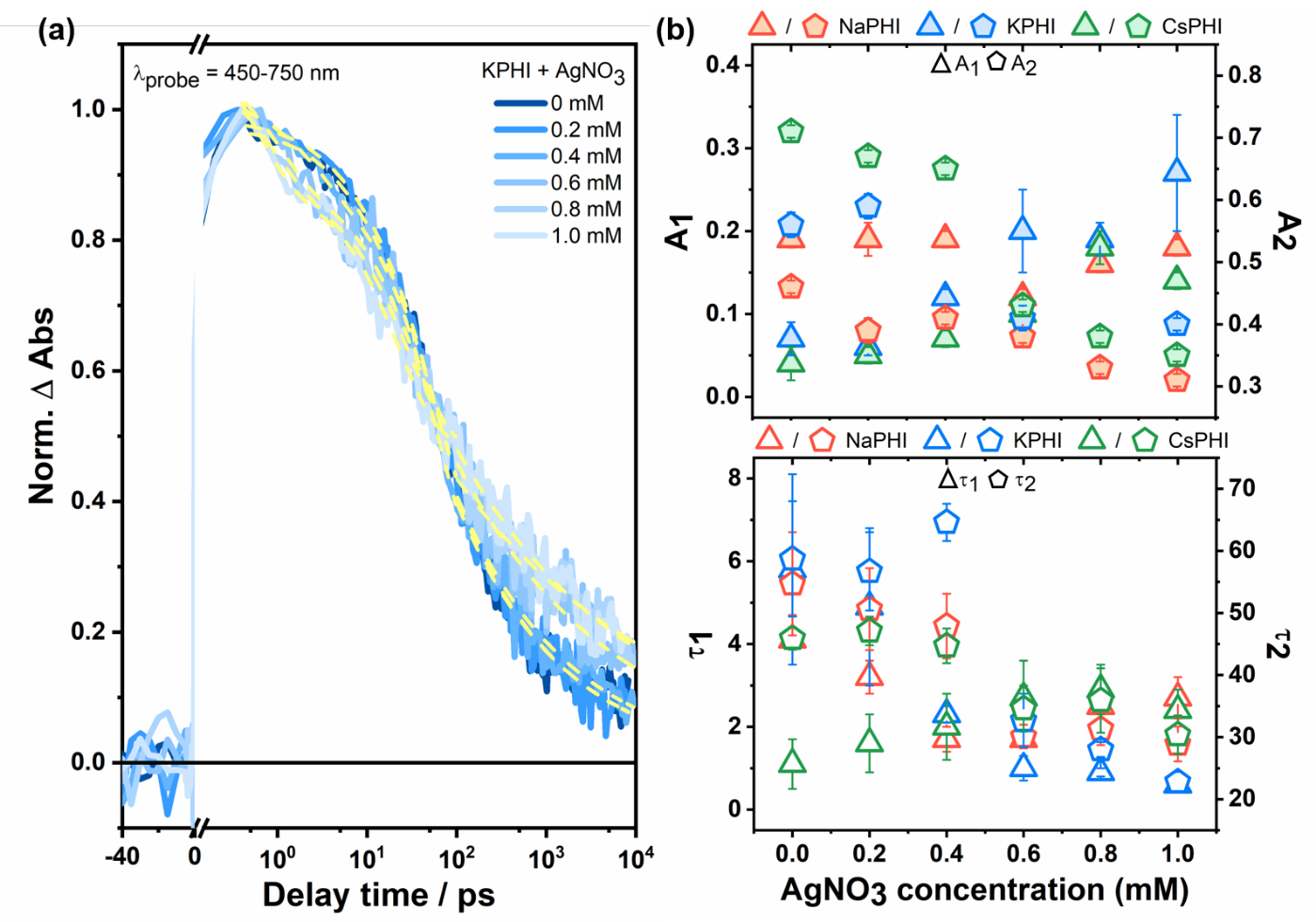


**Figure S12.** (a) Charge recombination kinetics of KPHI (concentration 1 g L^‒1^) probed from 450-750 nm with variation of AgNO_3_ concentrations from 0.2 mM to 1 mM. The yellow dashed lines represent biexponential and power law fitting. (b) Change of amplitudes ($A_{1}$ and $A_{2}$) obtained from the biexponential fit within 100 ps of KPHI (concentration 1 g L^‒1)^ with varying AgNO_3_ concentrations (upper panel) and change of characteristics time constants ($\tau_{1}$ and $\tau_{2}$) obtained from the biexponential fit within 100 ps of KPHI (concentration 1 g L^‒1^) with varying AgNO_3_ concentrations (lower panel). Each sample was measured in triplicate, and the error bars are given as ±σ (σ is the standard deviation of the fitted parameter obtained from the nonlinear regression using OriginLab software).

Overall, AgNO_3_ at lower concentration (< 0.4 mM) does not significantly impact the TA decay kinetics on the fs-ns timescale, but in the presence of higher AgNO_3_ concentration, the change is pronounced. However, it is noteworthy that increasing the concentration of AgNO_3_ might lead to an increase in the ionic strength, which in turn will reduce the Ag^+^ to Ag^0^, and tiny silver particles will be formed. This can further cause gelation and precipitation of KPHI that ultimately might influence the TA behaviour. The abrupt changes in the characteristic time constants and amplitudes while analysing the decay kinetics of KPHI in the presence of AgNO_3_ (0.4 mM versus 0.6 mM) can potentially be attributed to the altered TA decay as a result of an increase in ionic strength in the presence of a higher concentration of AgNO_3_.


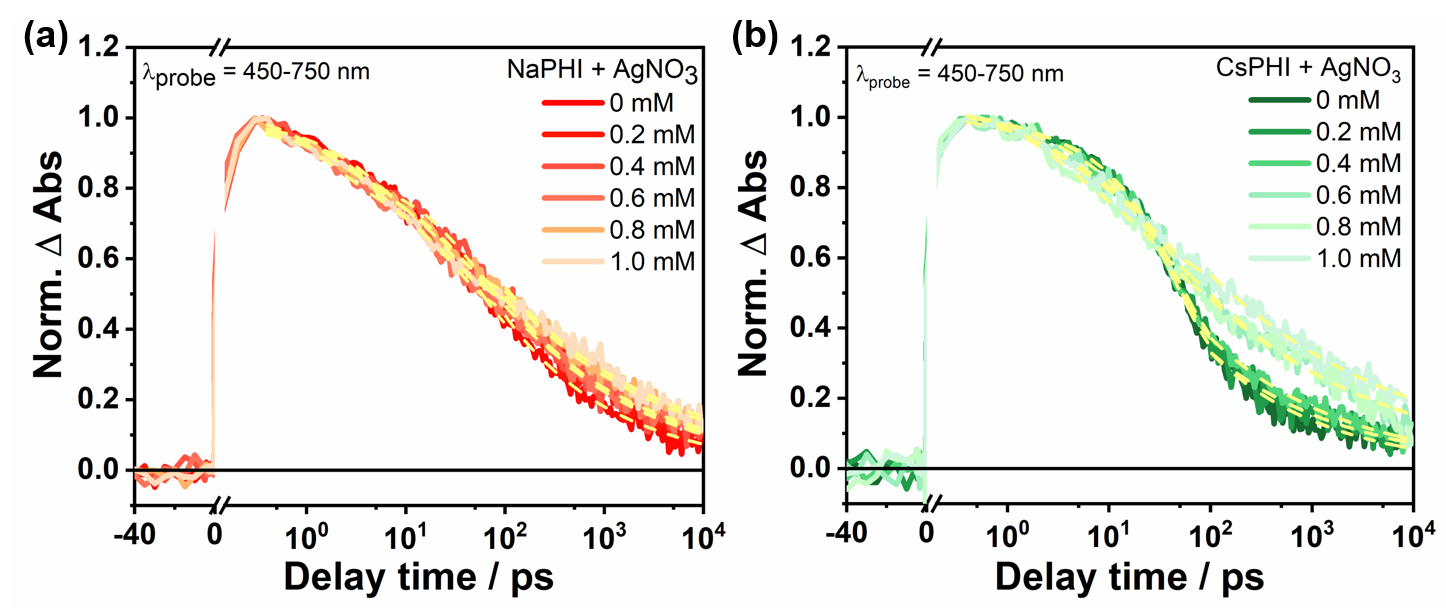


**Figure S13.** Normalized fs-transient absorption decay kinetics of (a) aqueous NaPHI and (b) aqueous CsPHI with various concentrations of AgNO_3_ (0 to 1 mM) as an electron quencher.

A similar AgNO_3_-induced behaviour is observed for NaPHI and CsPHI (**Figure S9**). In case of NaPHI (**Figure S9a**, Supporting Information), exciton decays with $\tau_{1}^{Na}$ being in the range of 1.7 to 4.1 ps at different AgNO_3_ concentrations, while $\tau_{2}^{Na}$ vary between 54.7 and 47.9 ps in the presence of 0-0.4 mM AgNO_3_ concentration and 31.3 to 28.9 ps with higher concentrations of AgNO_3_. Unlike the other two PHIs, CsPHI (**Figure S9b**) shows a minute increase of $\tau_{1}^{Cs}$ from 1.1 to 2.7 ps with increasing AgNO_3_ concentrations. However, $\tau_{2}^{Cs}$ appears to be in the same fashion as observed for $\tau_{2}^{Na}$ or $\tau_{2}^{K}$, *i.e.*, within 0.4 mM AgNO_3_ concentration, it ranges between 47.1 to 44.7 ps, and a characteristic decay time between 35.9 and 30.4 ps is observed for CsPHI with higher AgNO_3_ concentrations. Concerning the deep-trap related kinetics in Na-, and CsPHI, a decrease in the $\beta^{Na}$and $\beta^{Cs}$ are evident with increasing AgNO_3_ concentrations. $\beta^{Na}$ remains almost similar (0.36 ± 0.03) in the presence of lower AgNO_3_ concentrations ($\leq$ 0.4 mM), while a change in $\beta^{Na}$ is visible (0.28 ± 0.02) upon increasing AgNO_3_ concentrations within 0.6 mM and 1 mM. CsPHI also seems to exhibit similar observations, with a contrast in the exponent range of $\beta^{Cs}$ (0.35 ± 0.03) in the presence of low concentrations of AgNO_3_ ($\leq$ 0.4 mM) versus 0.23 ± 0.02 in presence of higher AgNO_3_ concentrations (**Table S4**). A detailed comparison among the decay kinetics fit parameters involving all the PHIs in the presence of AgNO_3_ is shown in **Figure S8b** and **Table S4**.

**Table S4.** Fit parameter values for the fs-transient absorption decay kinetics of NaPHI (top), KPHI (middle) and CsPHI (bottom) (concentration of 1 g L^–1^) with different AgNO_3_ concentrations ranging from 0 to 1 mM. The TA kinetics of all the PHIs can be biexponentially fitted within the first 100 ps by $I=A_{1}e^{-t/\tau_{1}}+A_{2}e^{-t/\tau_{2}}+I_{0}$ and at the longer delay times, the decay kinetics are represented by a power-law relationship: $I\propto t^{-\beta}$, where I denotes the TA signal at a specific probe wavelength at a specific delay time, $\tau_{1}$ and $\tau_{2}$ are two characteristic time constants, $A_{1}$ and $A_{2}$ are the respective amplitudes associated with $\tau_{1}$ and $\tau_{2}$, $I_{0}$ represents the approximation for the fraction of excitations within 100 ps that end up in deep traps, and β is the exponent of the power-law. Each sample was measured in triplicate, and the errors are given as *±σ* (*σ* is the standard deviation of the fitted parameter obtained from the nonlinear regression using OriginLab software)*.*

|  | NaPHI with different AgNO_3_ concentrations | | | | | |
| --- | --- | --- | --- | --- | --- | --- |
|  | 0 mM | 0.2 mM | 0.4 mM | 0.6 mM | 0.8 mM | 1.0 mM |
| $I_{0}$ | 0.34 ± 0.03 | 0.41 ± 0.02 | 0.43 ± 0.02 | 0.44 ± 0.01 | 0.49 ± 0.01 | 0.48 ± 0.01 |
| $A_{1}$ | 0.19 ± 0.01 | 0.19 ± 0.02 | 0.19 ± 0.01 | 0.12 ± 0.01 | 0.16 ± 0.01 | 0.18 ± 0.01 |
| $\tau_{1}$ | 4.1 ± 0.6 | 3.2 ± 0.4 | 1.7 ± 0.3 | 1.7 ± 0.2 | 2.5 ± 0.4 | 2.7 ± 0.5 |
| $A_{2}$ | 0.46 ± 0.01 | 0.39 ± 0.01 | 0.41 ± 0.02 | 0.38 ± 0.01 | 0.33 ± 0.01 | 0.31 ± 0.01 |
| $\tau_{2}$ | 54.7 ± 8.3 | 50.6 ± 6.6 | 47.9 ± 5.2 | 30.1 ± 1.9 | 31.3 ± 2.6 | 28.9 ± 2.8 |
| β | 0.39 ± 0.01 | 0.34 ± 0.01 | 0.33 ± 0.01 | 0.30 ± 0.01 | 0.28 ± 0.01 | 0.26 ± 0.01 |

|  | KPHI with different AgNO_3_ concentrations | | | | | |
| --- | --- | --- | --- | --- | --- | --- |
|  | 0 mM | 0.2 mM | 0.4 mM | 0.6 mM | 0.8 mM | 1.0 mM |
| $I_{0}$ | 0.33 ± 0.04 | 0.33 ± 0.03 | 0.26 ± 0.02 | 0.48 ± 0.02 | 0.49 ± 0.01 | 0.49 ± 0.01 |
| $A_{1}$ | 0.07 ± 0.02 | 0.06 ± 0.01 | 0.12 ± 0.01 | 0.20 ± 0.05 | 0.19 ± 0.02 | 0.27 ± 0.07 |
| $\tau_{1}$ | 5.8 ± 2.3 | 4.9 ± 1.9 | 2.3 ± 0.2 | 1.0 ± 0.3 | 0.9 ± 0.1 | 0.6 ± 0.1 |
| $A_{2}$ | 0.56 ± 0.02 | 0.59 ± 0.02 | 0.65 ± 0.01 | 0.41 ± 0.02 | 0.38 ± 0.01 | 0.40 ± 0.01 |
| $\tau_{2}$ | 58.7 ± 9.3 | 56.7 ± 6.3 | 64.6 ± 3.0 | 32.7 ± 4.3 | 28.0 ± 1.2 | 22.8 ± 1.6 |
| β | 0.37 ± 0.01 | 0.38 ± 0.01 | 0.35 ± 0.01 | 0.25 ± 0.01 | 0.19 ± 0.01 | 0.21 ± 0.01 |

|  | CsPHI with different AgNO_3_ concentrations | | | | | |
| --- | --- | --- | --- | --- | --- | --- |
|  | 0 mM | 0.2 mM | 0.4 mM | 0.6 mM | 0.8 mM | 1.0 mM |
| $I_{0}$ | 0.27 ± 0.01 | 0.30 ± 0.01 | 0.30 ± 0.02 | 0.48 ± 0.01 | 0.46 ± 0.02 | 0.54 ± 0.01 |
| $A_{1}$ | 0.04 ± 0.02 | 0.05 ± 0.01 | 0.07 ± 0.01 | 0.10 ± 0.02 | 0.18 ± 0.02 | 0.14 ± 0.01 |
| $\tau_{1}$ | 1.1 ± 0.6 | 1.6 ± 0.7 | 2.0 ± 0.8 | 2.7 ± 0.9 | 0.9 ± 0.6 | 2.4 ± 0.5 |
| $A_{2}$ | 0.71 ± 0.01 | 0.67 ± 0.01 | 0.65 ± 0.01 | 0.43 ± 0.01 | 0.38 ± 0.01 | 0.35 ± 0.01 |
| $\tau_{2}$ | 45.9 ± 1.8 | 47.1 ± 2.3 | 44.7 ± 2.8 | 34.6 ± 3.7 | 35.9 ± 5.2 | 30.4 ± 3.1 |
| β | 0.38 ± 0.02 | 0.33 ± 0.01 | 0.33 ± 0.01 | 0.25 ± 0.01 | 0.26 ± 0.01 | 0.22 ± 0.01 |


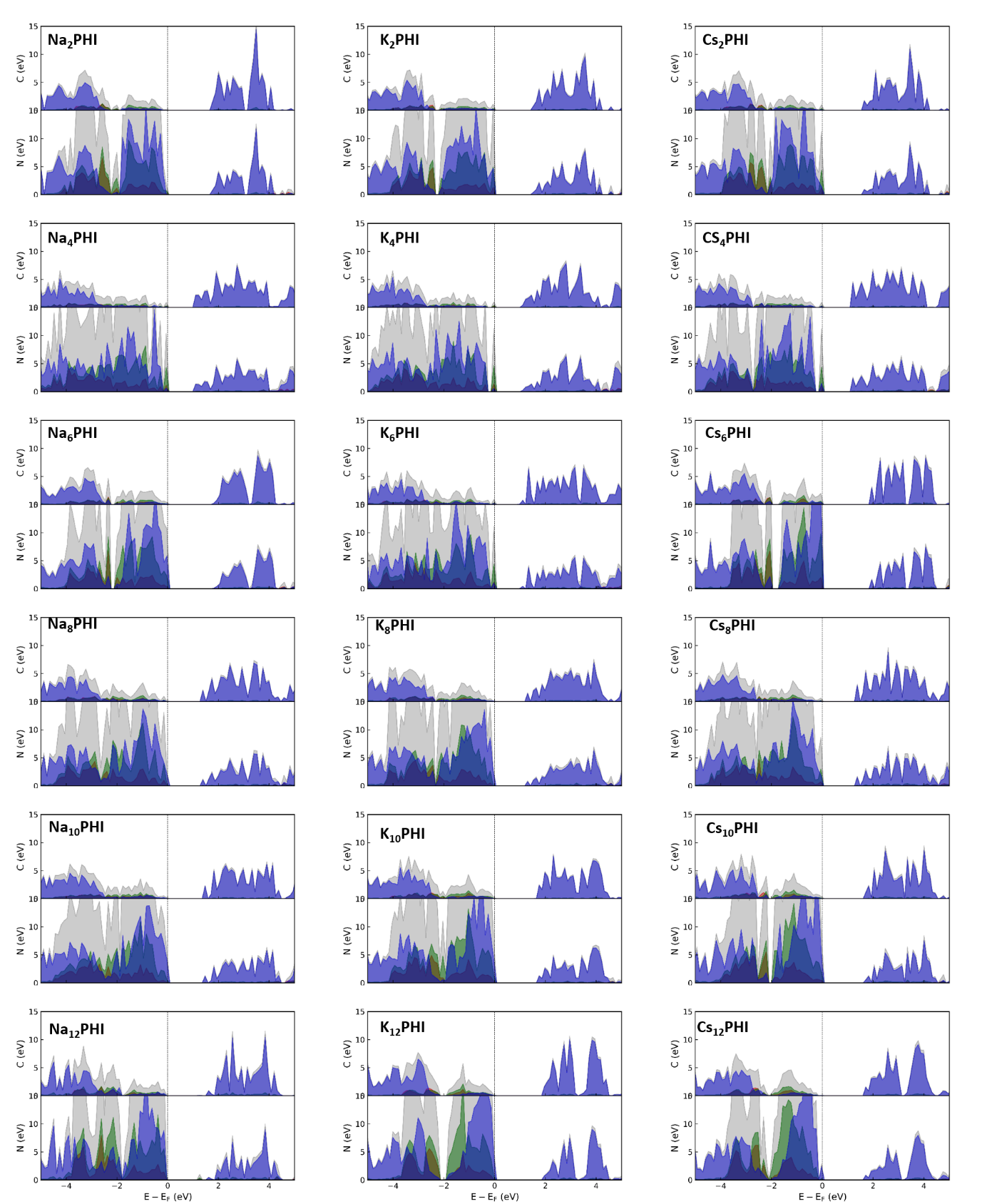


**Figure S14.** Projected density of states of s (red), $x_{z}$ (blue), $x_{x+y}$ (green) orbitals, and total density of states (gray) for C and N elements.


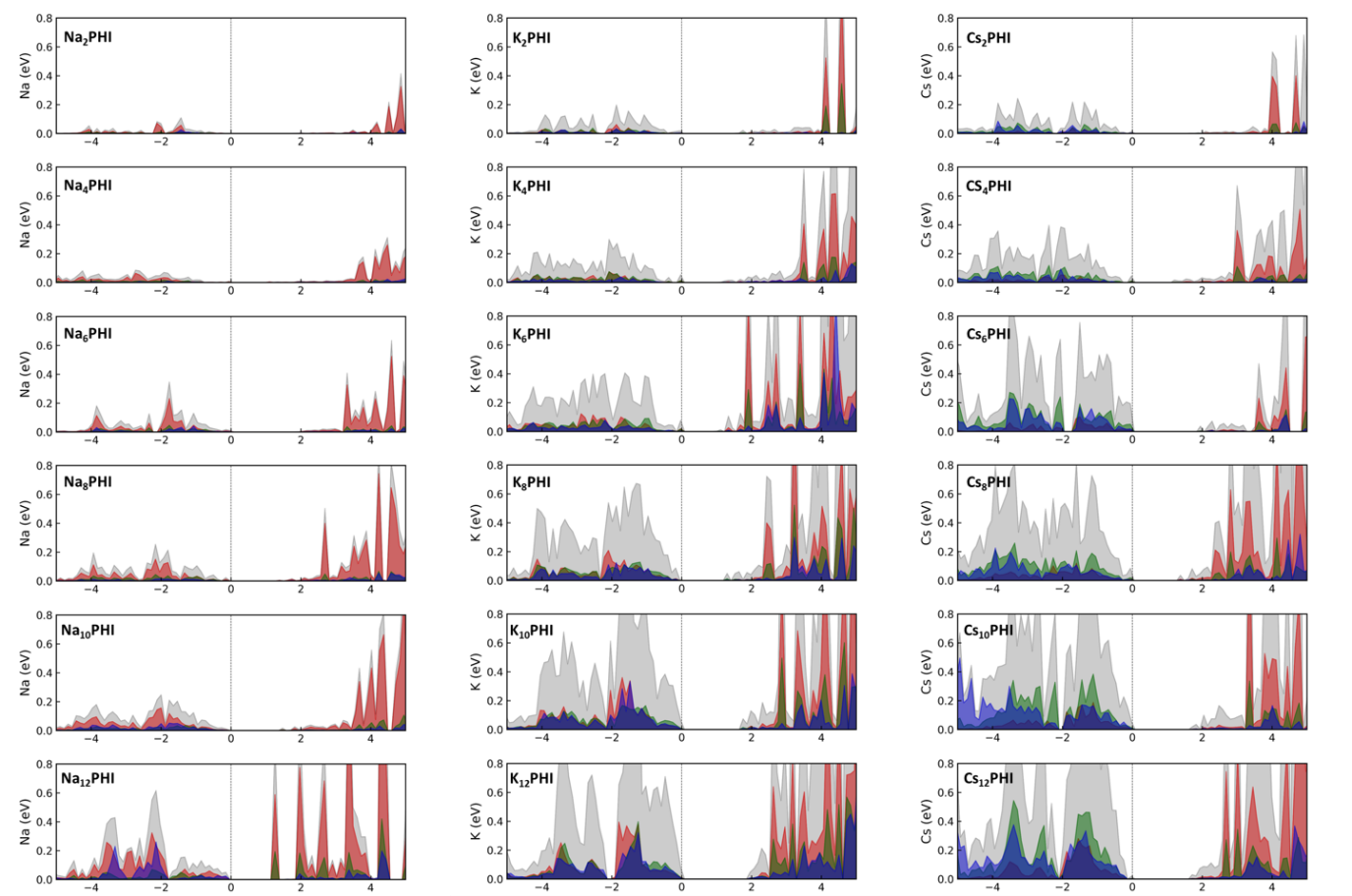


**Figure S15.** Projected density of states of s (red), $x_{z}$ (blue), $x_{x+y}$ (green) orbitals, and total density of states (gray) for the respective cations.


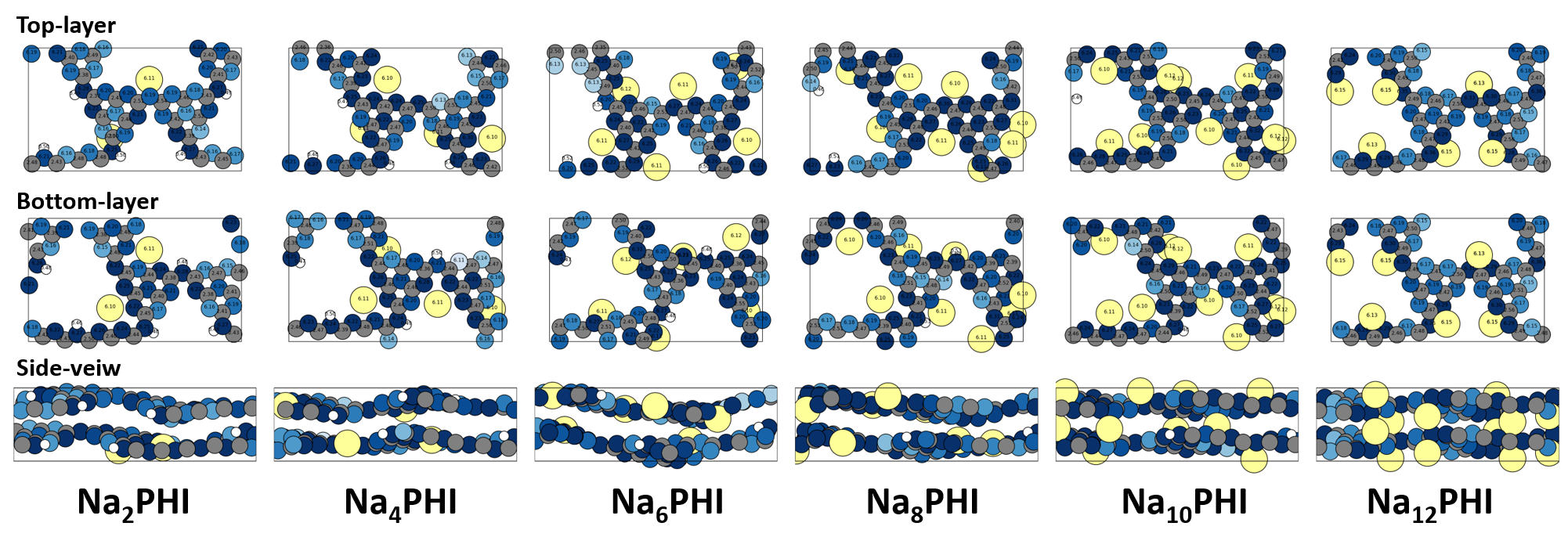


**Figure S16**. Bader charge analysis of NaPHI structures with varying Na content. Top-layer, bottom-layer, and side views are displayed separately to provide a clearer visualization of the evolving corrugation. All cation positions are distinctly represented to emphasize their placement within interstitial and interlayer sites. Element colors are assigned as follows: Na in pale yellow, C in gray, H in white, and N in a blue gradient-dark blue representing higher electron density and pale blue representing lower electron density-to illustrate the charge variations of nitrogen interacting with sodium.


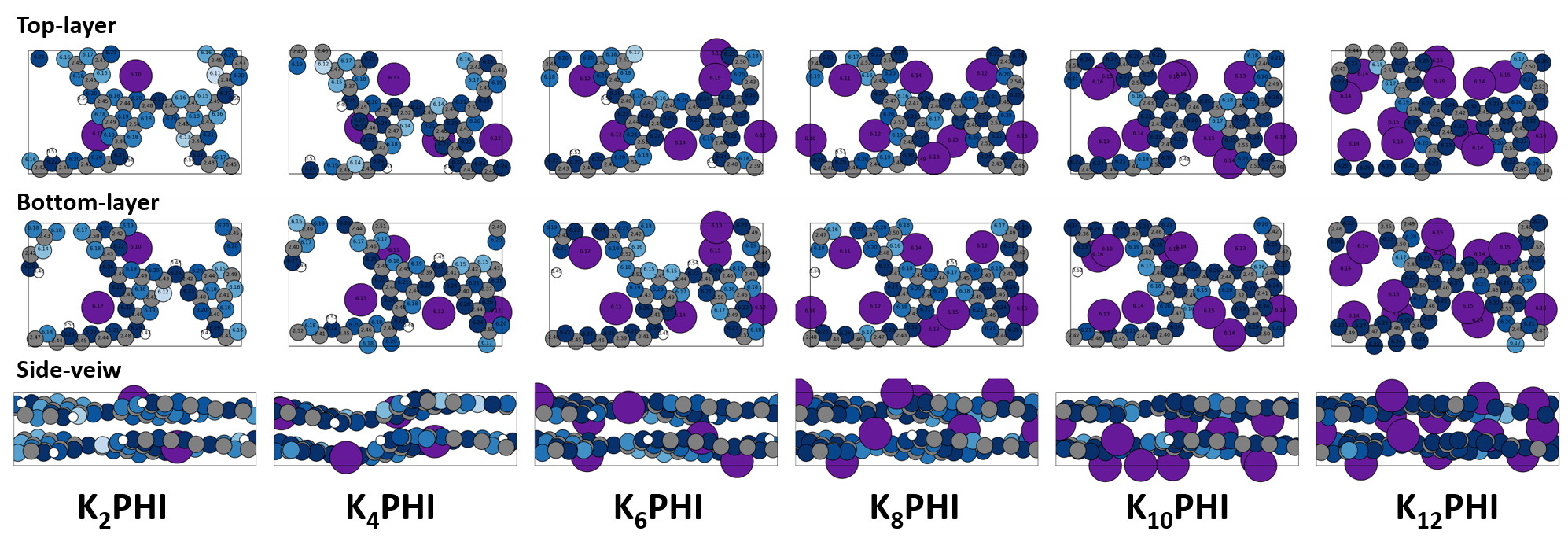


**Figure S17**. Bader charge analysis of KPHI structures with varying K content. Top-layer, bottom-layer, and side views are displayed separately to provide a clearer visualization of the evolving corrugation. All cation positions are distinctly represented to emphasize their placement within interstitial and interlayer sites. Element colors are assigned as follows: Na in pale yellow, C in gray, H in white, and N in a blue gradient-dark blue representing higher electron density and pale blue representing lower electron density-to illustrate the charge variations of nitrogen interacting with potassium.


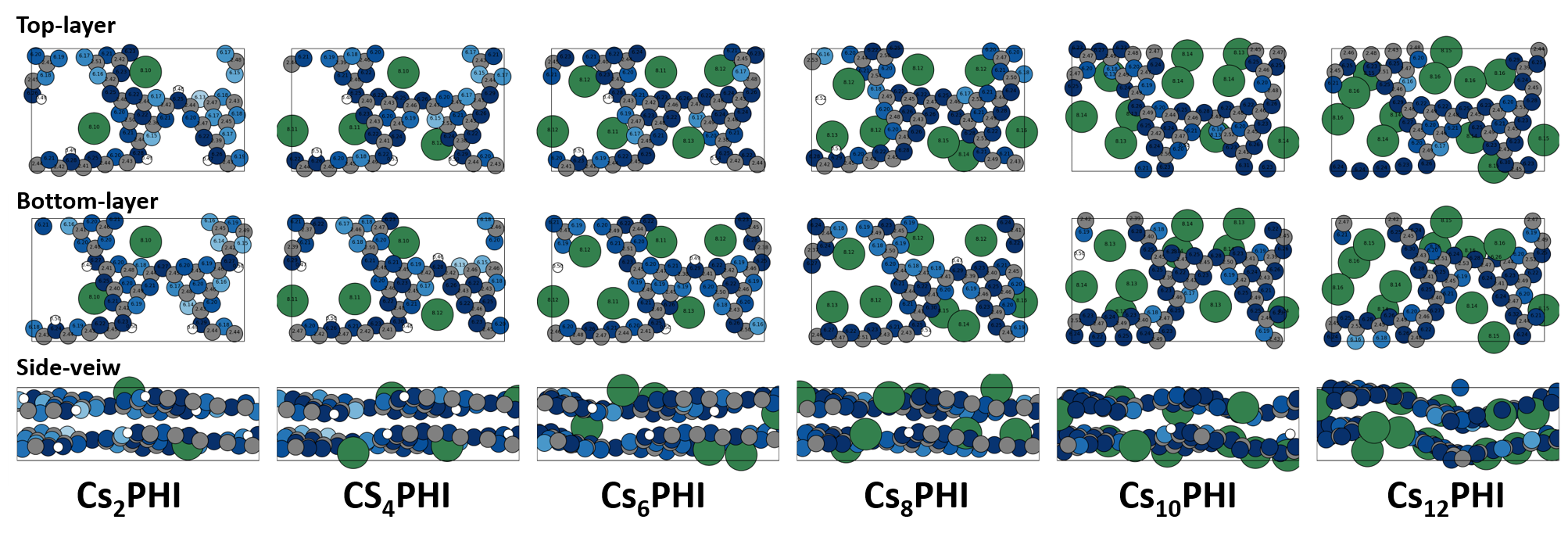


**Figure S18**. Bader charge analysis of CsPHI structures with varying Cs content. Top-layer, bottom-layer, and side views are displayed separately to provide a clearer visualization of the evolving corrugation. All cation positions are distinctly represented to emphasize their placement within interstitial and interlayer sites. Element colors are assigned as follows: Na in pale yellow, C in gray, H in white, and N in a blue gradient-dark blue representing higher electron density and pale blue representing lower electron density-to illustrate the charge variations of nitrogen interacting with cesium.


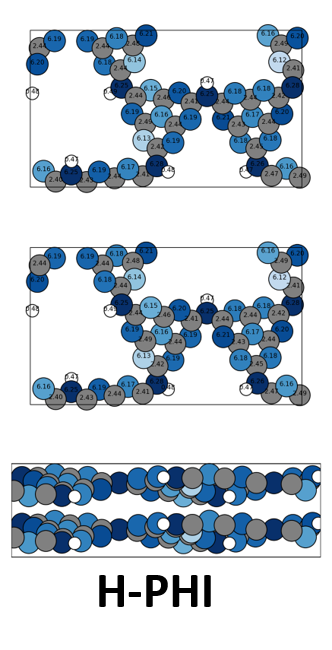


**Figure S19**. Bader charge analysis of PHI structures without any cation. Top-layer, bottom-layer, and side views are displayed separately to facilitate a clearer visualization. The color of elements set as follows: C in gray, H in white, and N in a blue gradient (dark blue indicating more electron density and pale blue indicating less electron density).


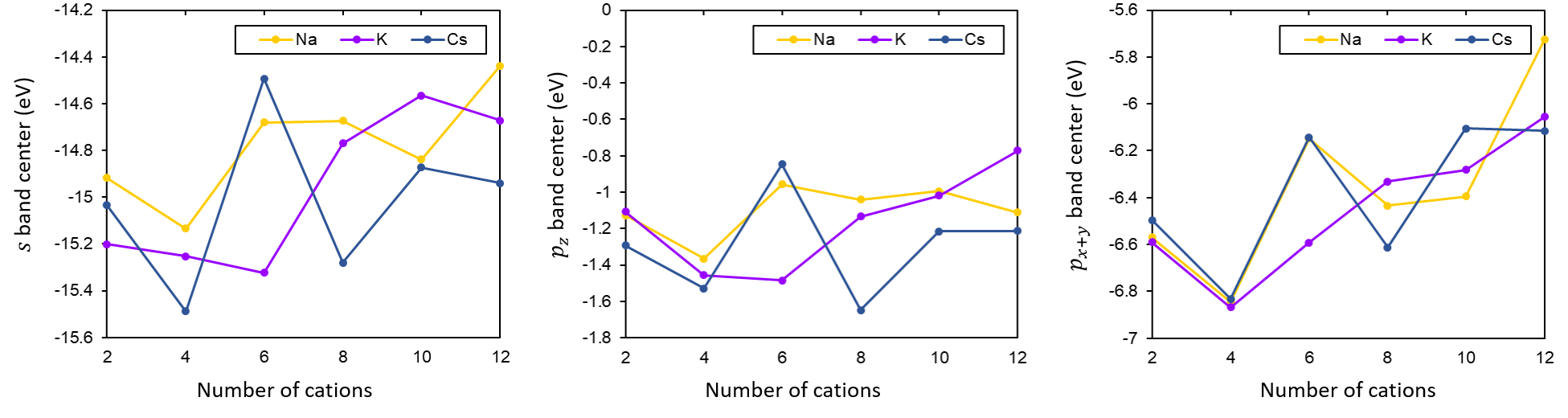


**Figure S20**. Effective band center energy of (a) $s$-band, (b) $p_{z}$-band and (c) $p_{x+y}$-band as function of varying cation contents.


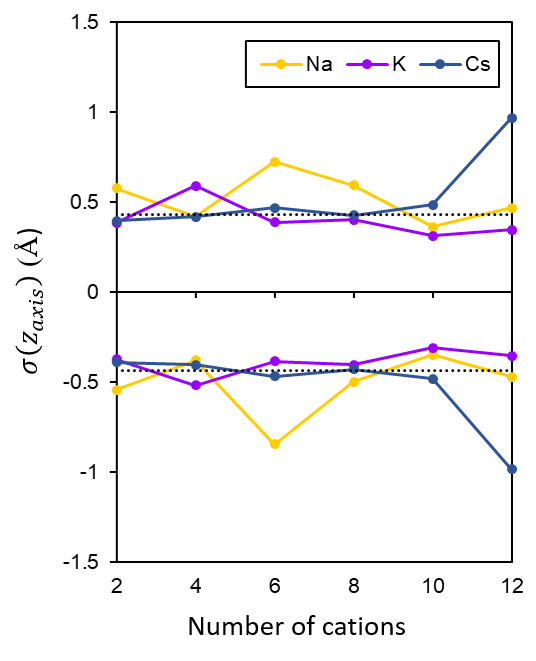


**Figure S21.** Standard deviations (with respect to genuine PHI structure) of z-coordinate for the C and N atoms calculated in the presence of various cations (K, Na and Cs).

**Table S5.** Effective band centers of the PHI without cations.

| Band center E (eV) | | | |
| --- | --- | --- | --- |
|  | $s$ | $p_{z}$ | $p_{x+y}$ |
| PHI | –14.07 | –0.74 | –6.12 |

**Table S6**. Standard deviation of the band centers as a function of cation contents.

| $\sigma(E)$ | $s$ | $p_{z}$ | $p_{x+y}$ |
| --- | --- | --- | --- |
| NaPHI | 0.22 | 0.13 | 0.35 |
| KPHI | 0.30 | 0.25 | 0.26 |
| CsPHI | 0.31 | 0.26 | 0.28 |

**References**

[1] a) G. Kresse, J. Hafner, *Phys. Rev. B* **1993**, 47, 558; b) G. Kresse, J. Hafner, *Phys. Rev. B* **1994**, 49, 14251; c) G. Kresse, J. Furthmüller, *Comput. Mater. Sci.* **1996**, 6, 15; d) G. Kresse, J. Furthmüller, *Phys. Rev. B* **1996**, 54, 11169; e) G. Kresse, [D. Joubert](https://journals.aps.org/search/field/author/D%20Joubert), *Phys. Rev. B* **1999**, 59, 1758.

[2] a) S. Grimme, J. Antony, S. Ehrlich, H. Krieg, *J. Chem. Phys.* **2010**, 132, 154104; b) [S. Grimme](https://onlinelibrary.wiley.com/authored-by/Grimme/Stefan), [S. Ehrlich](https://onlinelibrary.wiley.com/authored-by/Ehrlich/Stephan), [L. Goerigk](https://onlinelibrary.wiley.com/authored-by/Goerigk/Lars), *J. Comput. Chem.* **2011**, 32, 1456.

[3] J[. P. Perdew](https://journals.aps.org/search/field/author/John%20P%20Perdew), [K. Burke](https://journals.aps.org/search/field/author/Kieron%20Burke), [M. Ernzerhof](https://journals.aps.org/search/field/author/Matthias%20Ernzerhof), *Phys. Rev. Lett.* **1996**, **77**, 3865.

[4] H. Schlomberg, J. Kröger, G. Savasci, M. W. Terban, S. Bette, I. Moudrakovski, V. Duppel, F. Podjaski, R. Siegel, J. Senker, R. E. Dinnebier, C. Ochsenfeld, B. V. Lotsch, *Chem. Mater.* **2019**, 31, 7478.

[5] Y. Markushyna, P. Lamagni, C. Teutloff, J. Catalano, N. Lock, G. Zhang, M. Antonietti, A. Savateev, *J. Mater. Chem. A* **2019**, 7, 24771.
